# Supplementary material for: Coronaviruses reprogram the tRNA epitranscriptome to favor viral protein expression
Source: Nat Commun. 2026 Feb 19;17:2944. doi: 10.1038/s41467-026-69700-w (PMC13031925; doi:10.1038/s41467-026-69700-w)

Supplementary Figure 1

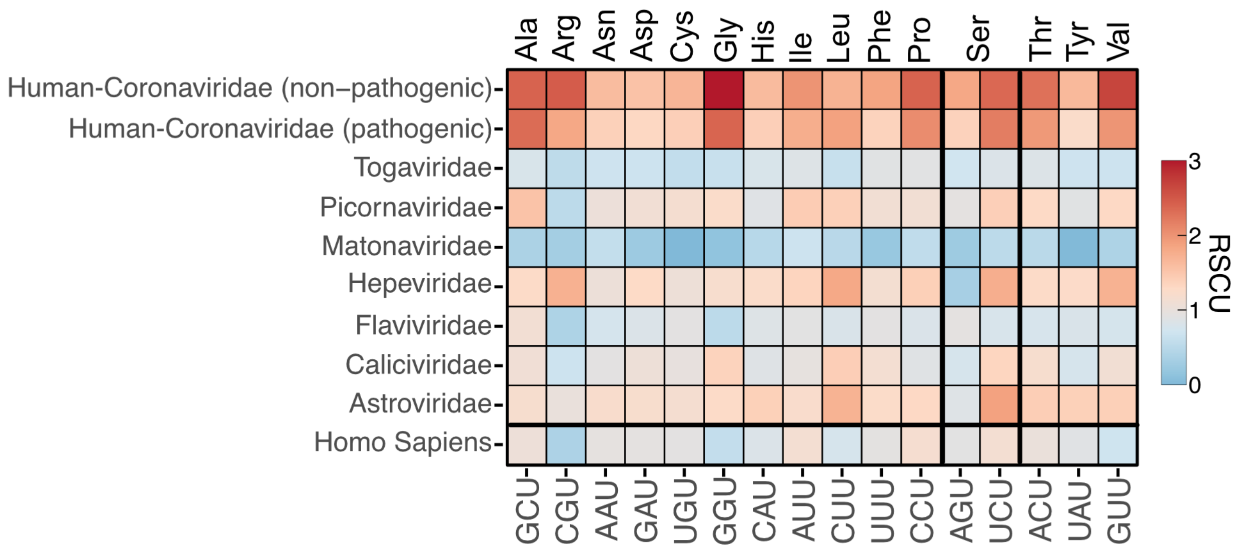

Supplementary Figure 1: RSCU analysis of U-ending codons in (+)RNA viruses.
Relative Synonymous Codon Usage (RSCU) analysis of U-ending codons in coding sequences of (+)RNA viruses, grouped by viral family, and *Homo sapiens*. Coding sequences were retrieved from the NCBI database.

Supplementary Figure 2

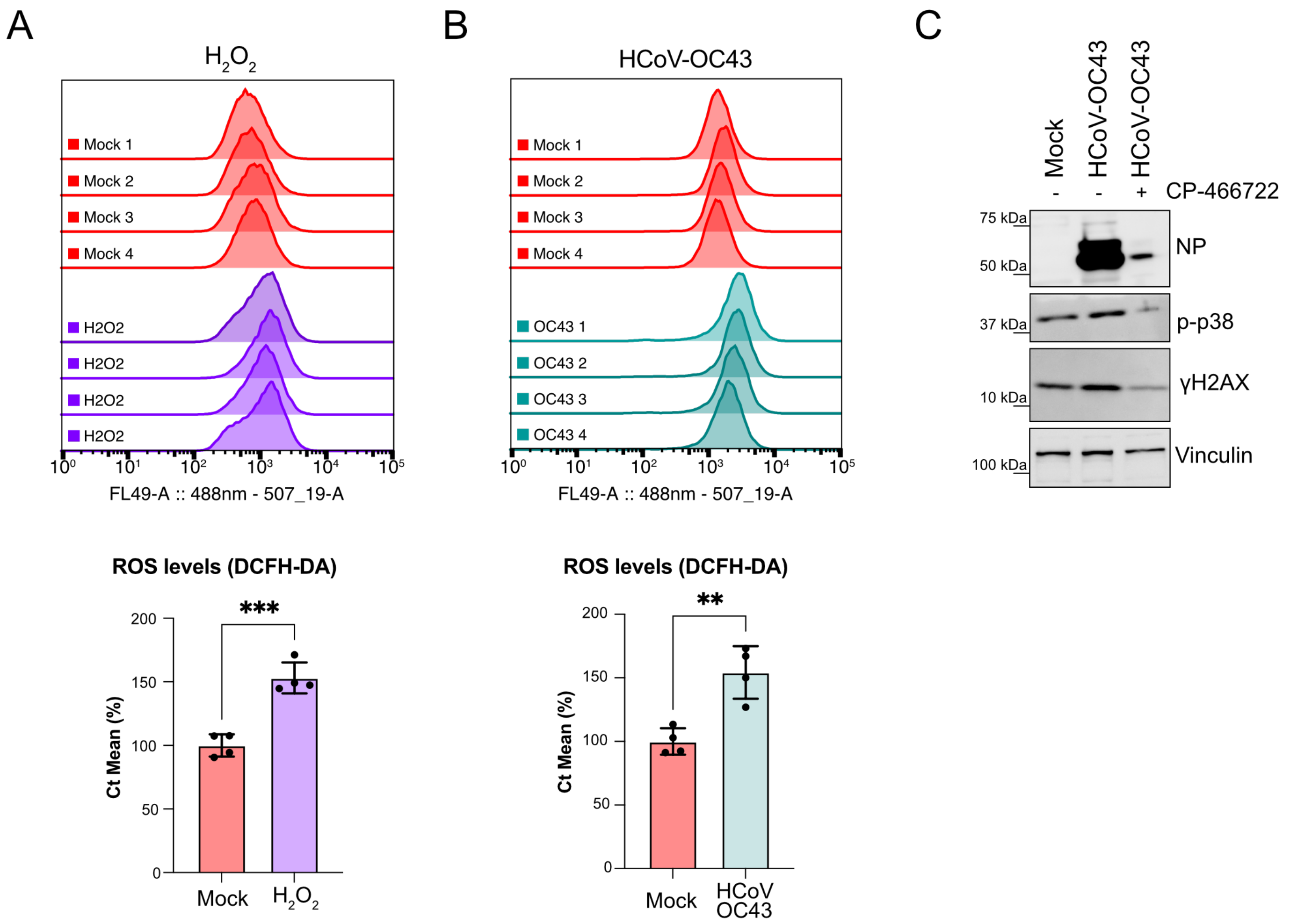

Supplementary Figure 2: Coronavirus infection induces ROS and requires DDR for replication.

(**A–B**) Mean fluorescence intensity (MFI) of intracellular ROS levels measured by flow cytometry using the DCFH-DA assay (10 µM) in A549 cells treated with H_2O_2 (1 mM, 30 min; positive control) (A) or infected with HCoV-OC43 (MOI 0.1, 48 h) (B), relative to mock-treated or mock-infected controls (n = 4). Statistical significance was assessed using an unpaired, two-sided t-test. Exact p-values are p = 0.0006 for A and p = 0.0073 for B. Data are presented as mean ± s.d. (**C**) Immunoblot analysis of viral NP protein expression and p-p38 phosphorylation in A549 cells infected with HCoV-OC43 (MOI 0.1, 48 h) and treated throughout the experiment with the ATM inhibitor CP-466722 (10 µM). Data shown are representative of three independent biological replicates (n = 3).

Supplementary Figure 3

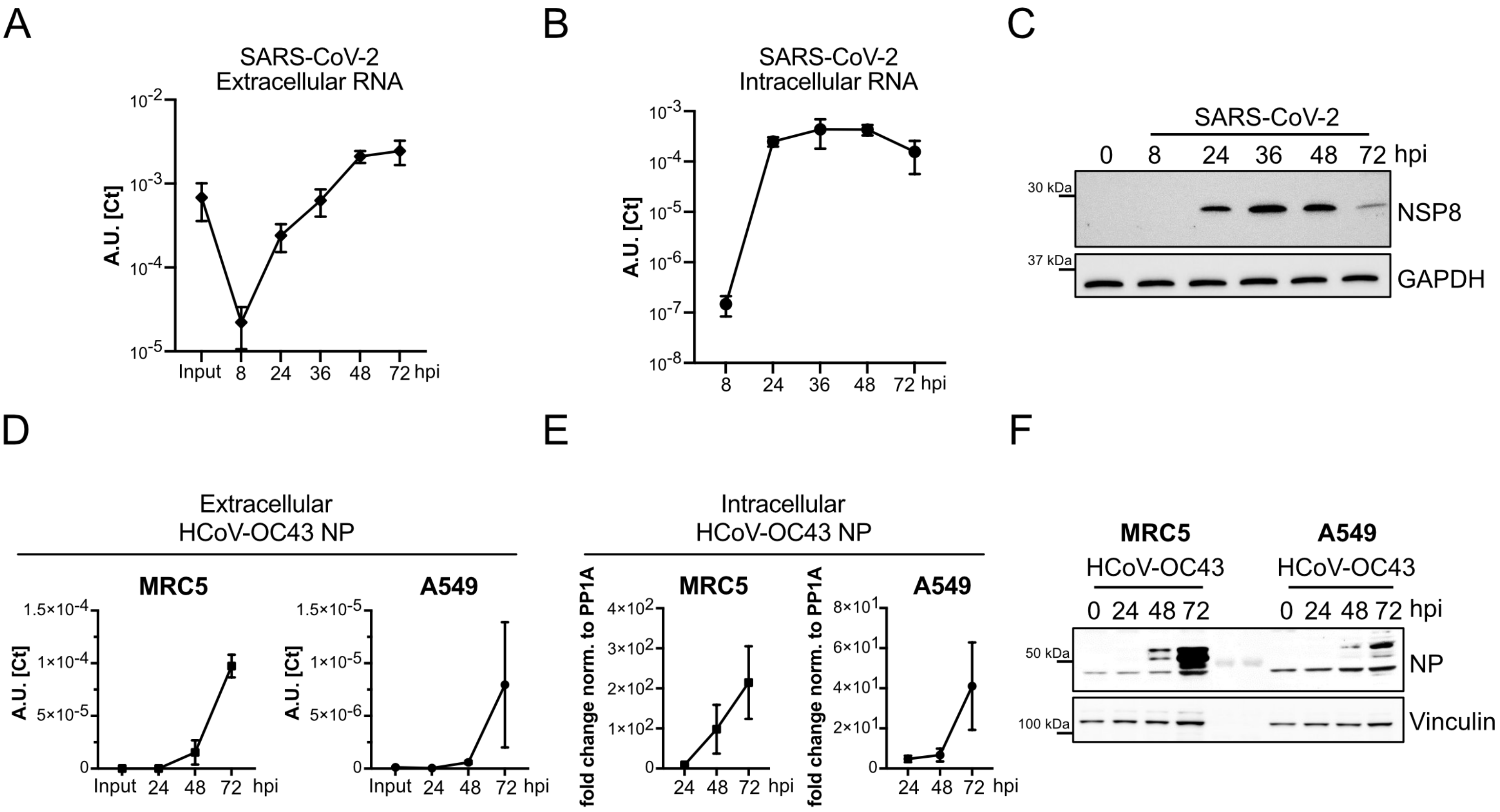

Supplementary Figure 3: **Coronavirus kinetics in lung cells.**

(**A-B**) qPCR analysis of SARS-CoV-2 extracellular and intracellular RNA extracted from Calu3-infected cells at the indicated times post-infection (n=3) using N as a target gene. (**C**) Western blot analysis of SARS-CoV-2 Non-structural protein 8 (NSP8) viral protein at 8, 24, 36 and 48 hours post-infection. (**D-E**) qPCR analysis of HCoV-OC43 extracellular and intracellular RNA extracted from MRC5-infected cells and A549-infected cells at the indicated times post-infection (n=3) using N as a target gene. (**F**) Western blot analysis of HCoV-OC43 N-viral protein at 24, 48 and 72 hours post-infection in MRC5-infected cells and A549-infected cells. Data shown are representative of three independent biological replicates (n = 3).

Supplementary Figure 4

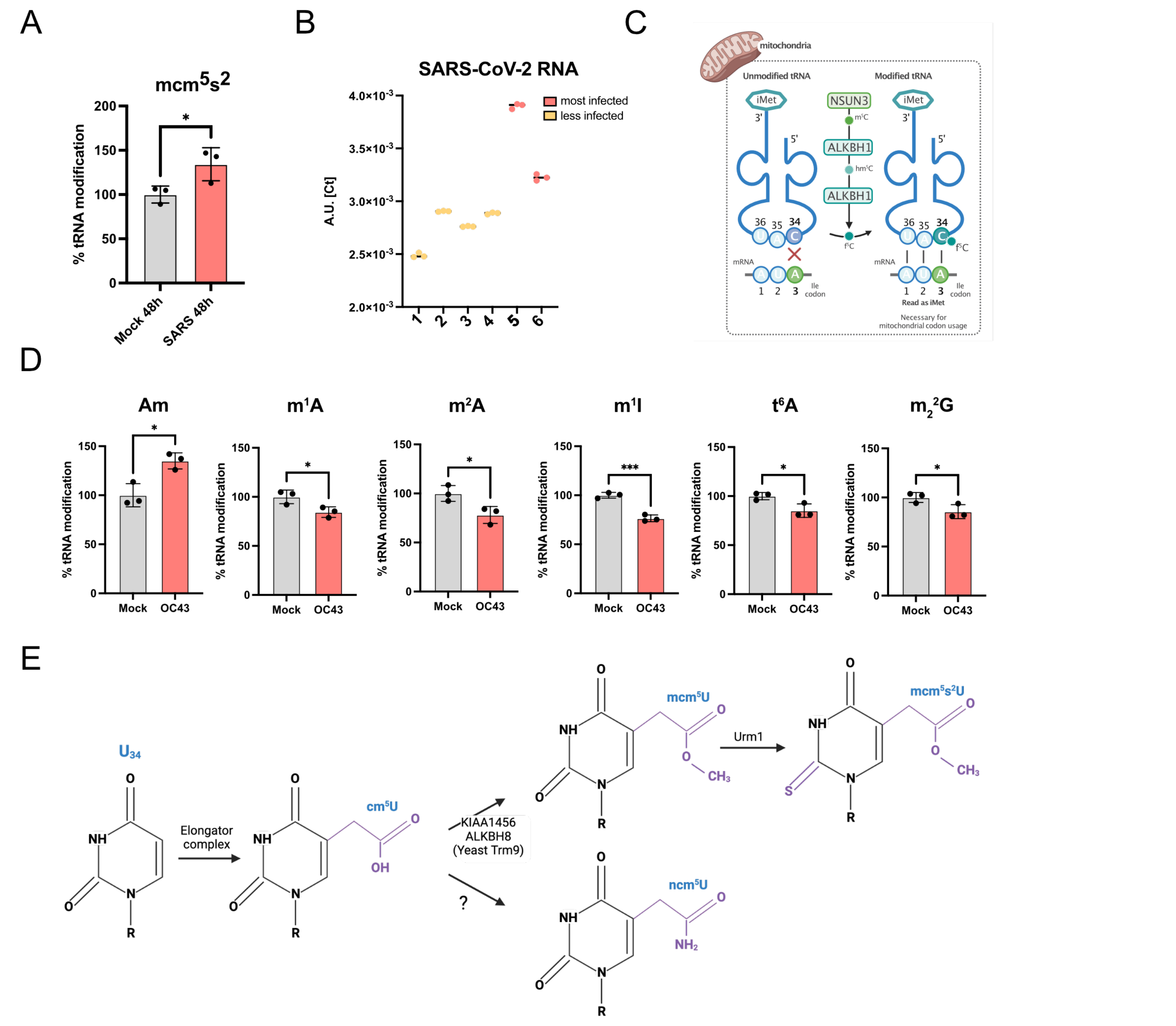

Supplementary Figure 4: tRNA modification analysis in coronaviruses infected cells.

(**A**) Quantification of mcm^{5}s^{2}U modification levels measured by LC–MS/MS in SARS-CoV-2–infected cells at 48 h post-infection relative to mock-infected controls (n = 3). Statistical significance was assessed using an unpaired, two-sided t-test (p = 0.0476). Data are presented as mean ± s.d. (**B**) Quantification of intracellular SARS-CoV-2 RNA levels by qPCR in hamster lung samples (n = 6), using the viral N gene as target. (C) Schematic representation of the NSUN3–ALKBH1 pathway responsible for tRNA-iMet modification. NSUN3 catalyzes the formation of m^{5}C at position 34 of tRNA-iMet, which is subsequently oxidized by ALKBH1 to generate 5-hydroxymethylcytosine (hm^{5}C) and 5-formylcytosine (f^{5}C). (D) Quantification of tRNA modifications measured by LC–MS/MS in HCoV-OC43–infected cells relative to mock-infected controls (n = 3), related to Fig. 3A. Statistical significance was assessed using an unpaired, two-sided t-test. Exact p-values are: Am, p = 0.0134; m^{1}A, p = 0.0371; m^{2}A, p = 0.0325; m^{1}I, p = 0.0008; t^{6}A, p = 0.0298; m^{22}G, p = 0.0465. Data are presented as mean ± s.d. (E) Schematic representation of the mcm^{5}U/mcm^{5}s^{2}U modification pathway (modifications are shown in purple). Panels (C) and panel (E) were created in Biorender. Diez, J. (2026) https://BioRender.com/177y1q2 and https://BioRender.com/rx9ld2u.

Supplementary Figure 5

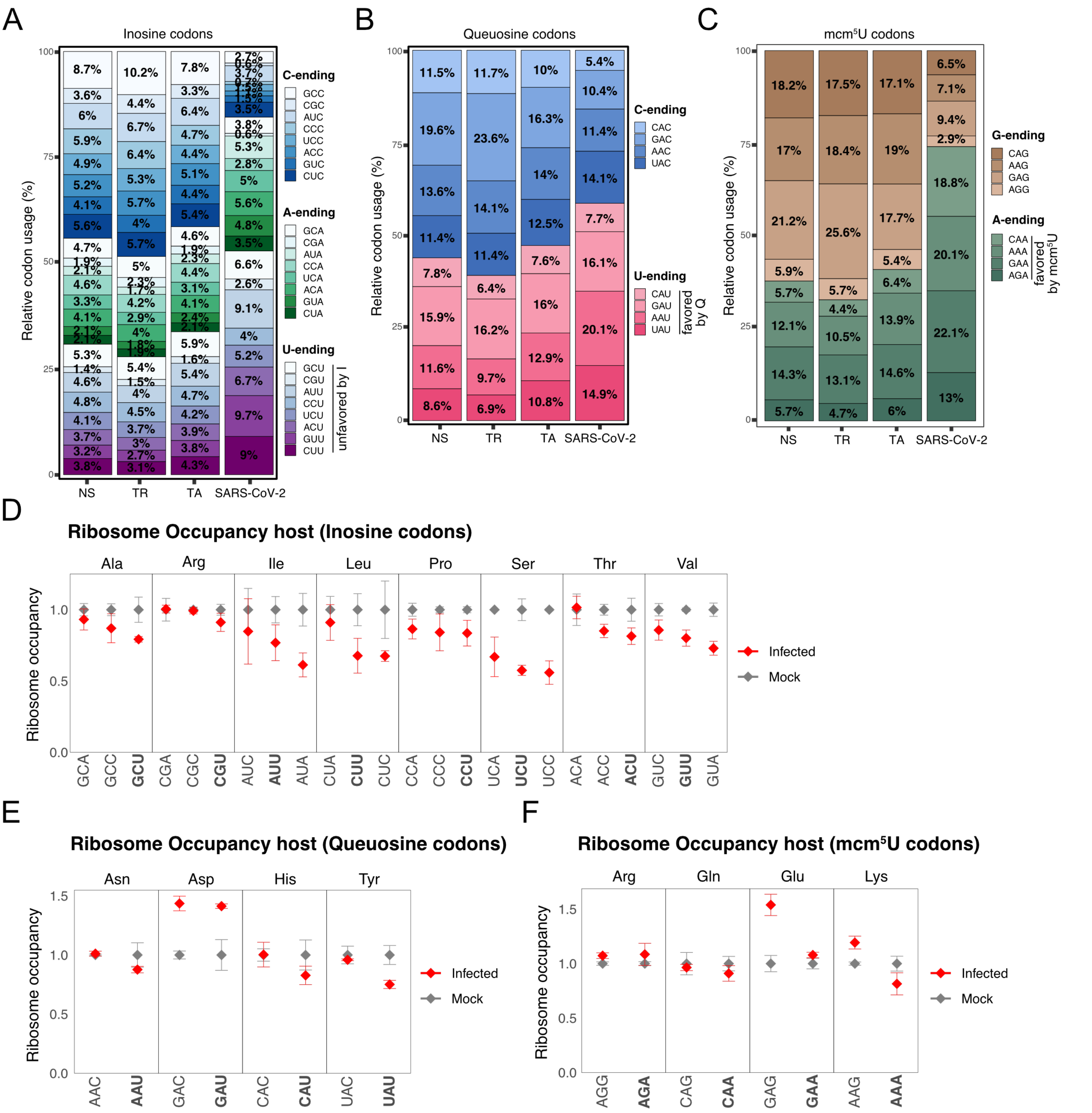

Supplementary Figure 5: Codons linked to I, Q and mcm^{5}U pathway are enriched in translationally activated genes during SARS-CoV-2 infection.

(**A**) Relative usage of C/A/U-ending codons recognized by I-tRNAs in translationally activated (TA) and translationally repressed genes (TR), compared to SARS-CoV-2 genes and translationally not changed genes (NS). (**B**) Relative usage of C/U-ending codons recognized by Q-tRNAs in translationally activated (TA) and translationally repressed genes (TR), compared to SARS-CoV-2 genes and translationally not changed genes (NS). (**C**) Relative usage of G/A-ending codons recognized by mcm^{5}U-tRNAs in translationally activated (TA) and translationally repressed genes (TR), compared to SARS-CoV-2 genes and translationally not changed genes (NS). Note that U-ending codons (panels A–B) and A-ending codons (panel A) are those preferred by coronaviruses. (**D-F**) Average host ribosome occupancy (P-site) in SARS-infected versus non-infected conditions for codons associated with Inosine (D), Queuosine (E), and mcm^{5}U (F) tRNA modifications. Codons enriched in SARS-CoV-2 genome are highlighted in bold.

Supplementary Figure 6

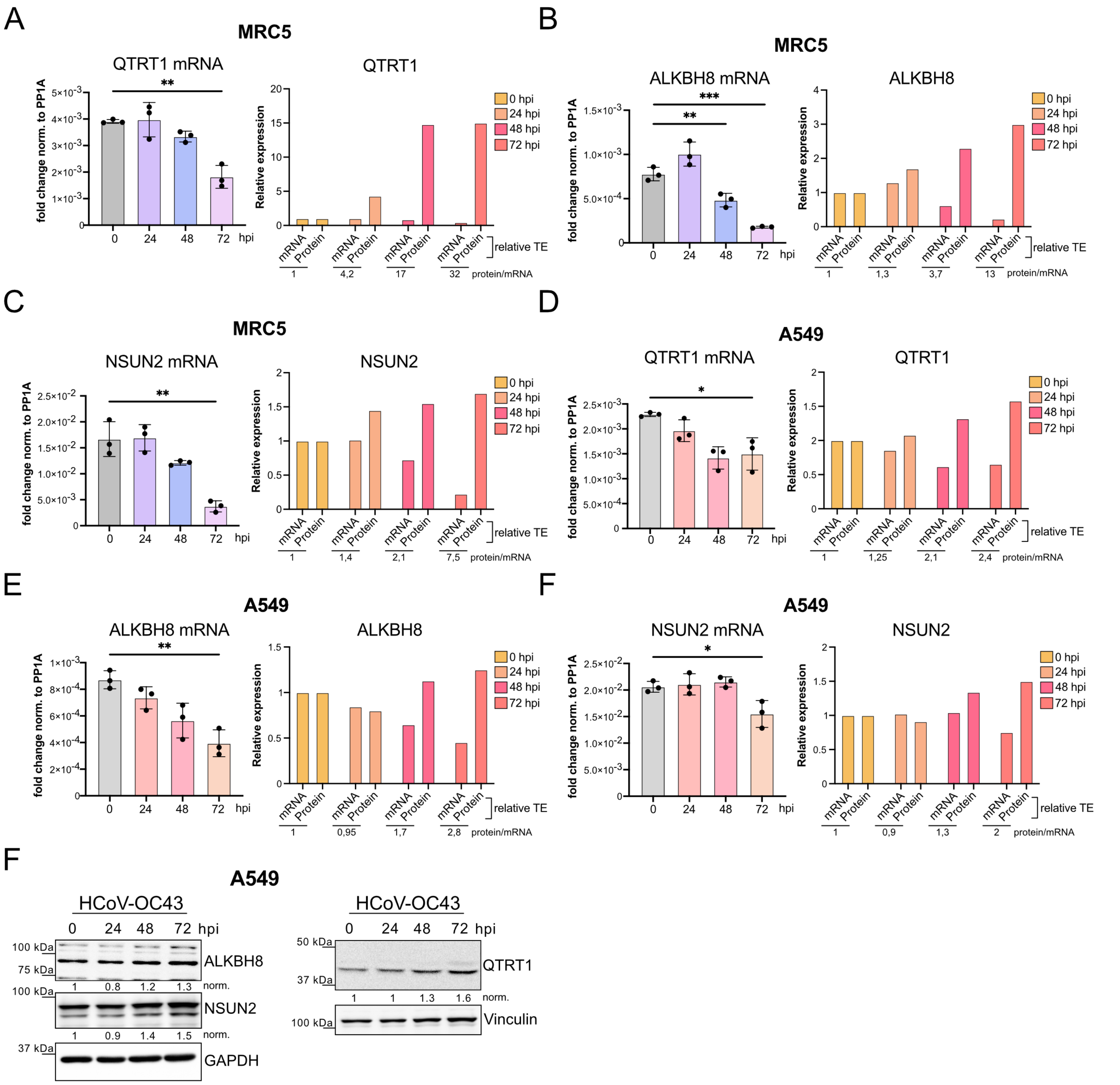

Supplementary Figure 6: Translational regulation of tRNA-modifying enzymes during HCoV-OC43 infection. (**A–C**) qPCR analysis of QTRT1 (A), ALKBH8 (B), and NSUN2 (C) mRNA levels and corresponding translation efficiency (TE; protein/RNA ratio) in MRC5 cells infected with HCoV-OC43 (MOI 0.1) for 0, 24, 48, and 72 h post-infection (hpi) (n = 3). Statistical significance relative to mock-infected controls was assessed using a two-sided, nonparametric Mann–Whitney U test. Exact p-values are: QTRT1, p = 0.0012 at 72 hpi; ALKBH8, p = 0.0092 at 48 hpi and p = 0.0002 at 72 hpi; NSUN2, p = 0.0031 at 72 hpi. (**D–F**) qPCR analysis and translation efficiency (TE; protein/RNA ratio) of QTRT1 (D), ALKBH8 (E), and NSUN2 (F) in A549 cells infected with HCoV-OC43 (MOI 0.1) for 0, 24, 48, and 72 hpi (n = 3). Statistical significance relative to mock-infected controls was assessed using a two-sided, nonparametric Mann–Whitney U test. Exact p-values are: QTRT1, p = 0.0138 at 72 hpi; ALKBH8, p = 0.0025 at 72 hpi; NSUN2, p = 0.0315 at 72 hpi. Translation efficiency (TE) was calculated as the ratio between protein levels (determined by western blot) and mRNA levels (determined by qPCR), with both values normalized to the corresponding mock-infected control. (**G**) Immunoblot analysis of QTRT1, ALKBH8, and NSUN2 protein levels in A549 cells infected with HCoV-OC43 (MOI 0.1) for 0, 24, 48, and 72 hpi. Western blot data used for TE calculations in MRC5 cells are shown in Figure 3. Data shown are representative of three independent biological replicates (n = 3).

Supplementary Figure 7

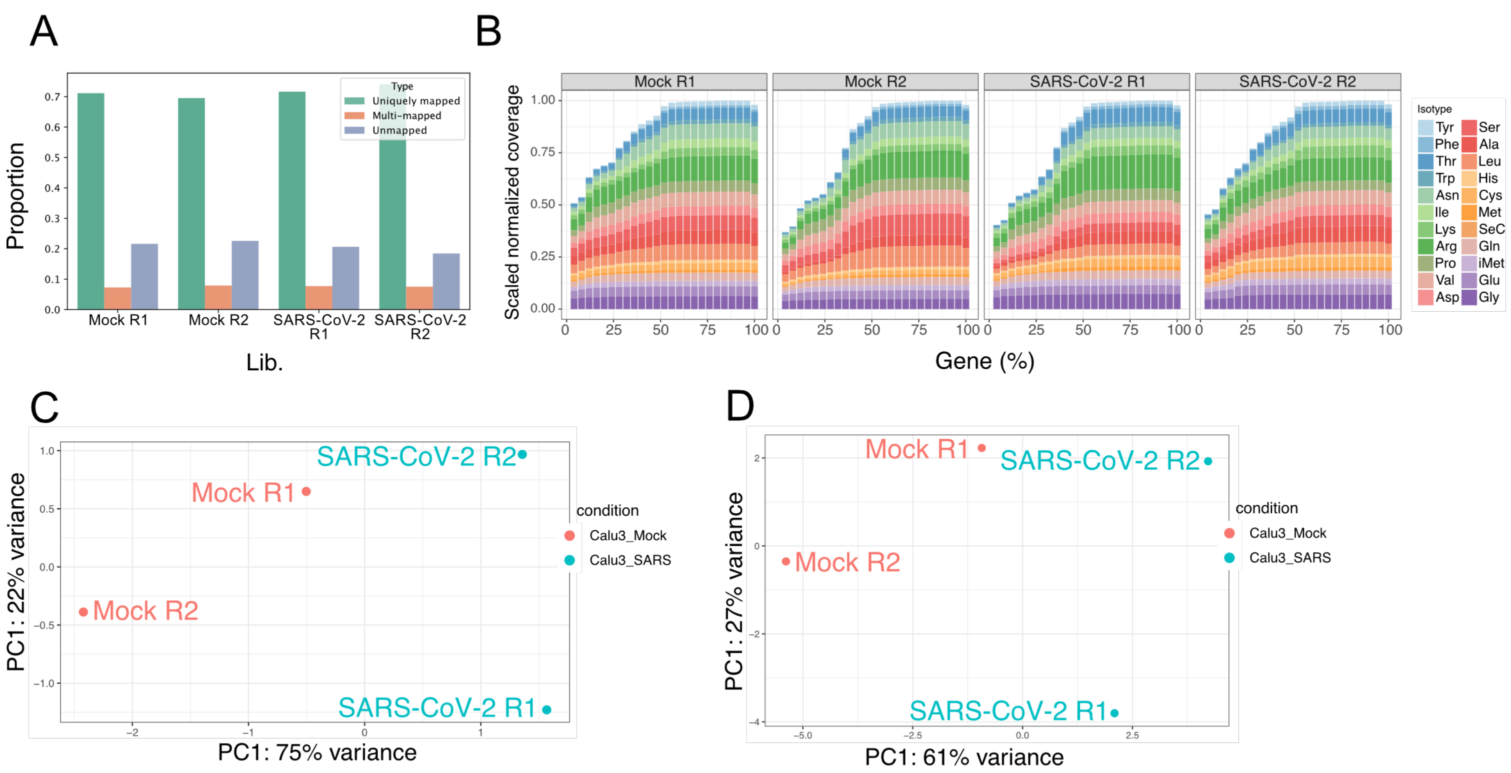

Supplementary Figure 7: Quality control of mim-tRNA-seq in SARS-CoV-2 infected cells.
(**A**) Alignment statistics for mim-tRNAseq datasets (SARS-CoV-2 in Calu3 cells) from the indicated conditions (n=2). Shown are the uniquely mapping, multi-mapped, and unmapped read proportions per library after realignment. (**B**) Quality control for tRNA coverage and full-length transcripts. Metagene plots of coverage per nuclear-encoded tRNA isotype ordered per sample by differences between 3′ and 5′ coverage (n=1). (**C-D**) Principal component analysis (PCA) plot using the first two principal components from tRNA isodecoder analysis (C) and tRNA anticodon analysis (D).

Supplementary Figure 8

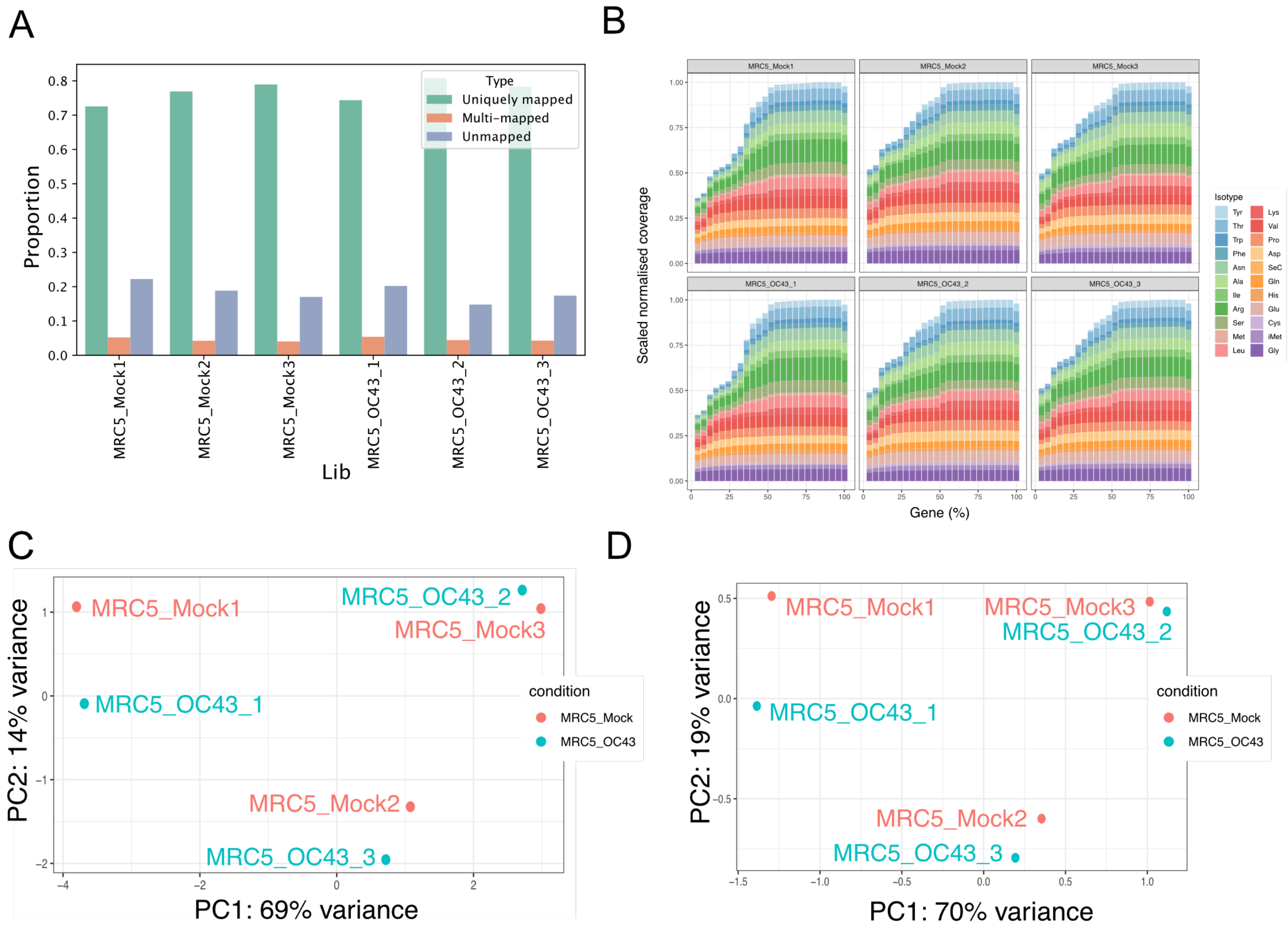

Supplementary Figure 8: Quality control of mim-tRNA-seq in HCoV-OC43 infected cells.

(**A**) Alignment statistics for mim-tRNAseq datasets (HCoV-OC43 in MRC5 cells) from the indicated conditions (n=3). Shown are the uniquely mapping, multi-mapped, and unmapped read proportions per library after realignment. (**B**) Quality control for tRNA coverage and full-length transcripts. Metagene plots of coverage per nuclear-encoded tRNA isotype ordered per sample by differences between 3′ and 5′ coverage (n=1). (**C-D**) Principal component analysis (PCA) plot using the first two principal components from tRNA isodecoder analysis (C) and tRNA anticodon analysis (D).

Supplementary Figure 9

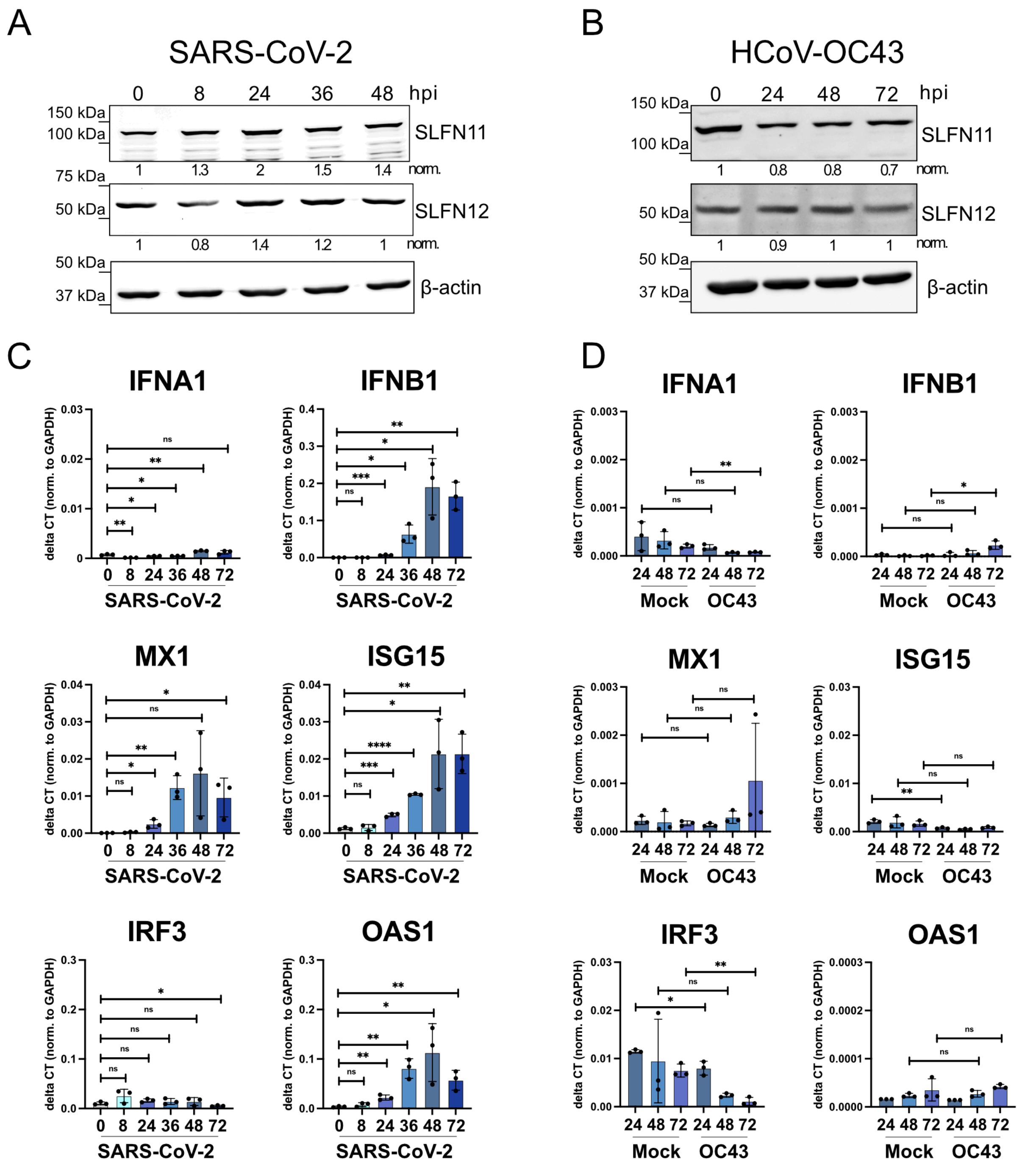

Supplementary Figure 9: SARS-CoV-2 infection triggers activation of SLFN11 and SLFN12.

(**A**) Immunoblot analysis of SLFN11 and SLFN12 protein levels in SARS-CoV-2–infected Calu3 cells (MOI 3) at the indicated times post-infection. β-actin was used as a loading control (n = 3). (**B**) Immunoblot analysis of SLFN11 and SLFN12 protein levels in HCoV-OC43–infected MRC5 cells (MOI 0.1) at the indicated times post-infection. β-actin was used as a loading control (n = 3). (**C**) qPCR analysis of interferon-related genes in SARS-CoV-2–infected Calu3 cells (MOI 3) at the indicated times post-infection, normalized to GAPDH expression (n = 3). Statistical significance relative to mock-infected controls was assessed using a two-sided, nonparametric Mann–Whitney U test. Exact p-values are: IFNA1: $p = 0.0011$ (8 h), $p = 0.0251$ (24 h), $p = 0.0213$ (36 h), $p = 0.0017$ (48 h), not significant (ns; $p = 0.0918$) at 72 h; IFNB1: ns at 8 h, $p = 0.0008$ (24 h), $p = 0.0112$ (36 h), $p = 0.0121$ (48 h), $p = 0.0016$ (72 h); IRF3: ns at 8–48 h, $p = 0.0456$ (72 h); MX1: ns at 8 h, $p = 0.0219$ (24 h), $p = 0.0027$ (36 h), ns at 48 h, $p = 0.0339$ (72 h); ISG15: ns at 8 h, $p = 0.0003$ (24 h), $p < 0.0001$ (36 h), $p = 0.0204$ (48 h), $p = 0.0028$ (72 h); OAS1: ns at 8 h, $p = 0.0040$ (24 h), $p = 0.0025$ (36 h), $p = 0.0316$ (48 h), $p = 0.0097$ (72 h). (D) qPCR analysis of interferon-related genes in HCoV-OC43–infected MRC5 cells (MOI 0.1) at the indicated times post-infection, normalized to GAPDH expression (n = 3). Statistical significance relative to mock-infected controls was assessed using a two-sided, nonparametric Mann–Whitney U test. Exact p-values are: IFNA1: ns at 24–48 h, $p = 0.0059$ (72 h); IFNB1: ns at 24–48 h, $p = 0.0130$ (72 h); IRF3: $p = 0.0148$ (24 h), ns at 48 h, $p = 0.0022$ (72 h); MX1: ns at all time points; OAS1: ns at all time points; ISG15: $p = 0.0048$ (24 h), ns at 48–72 h. Data shown are representative of three independent biological replicates (n = 3).

Supplementary Figure 10

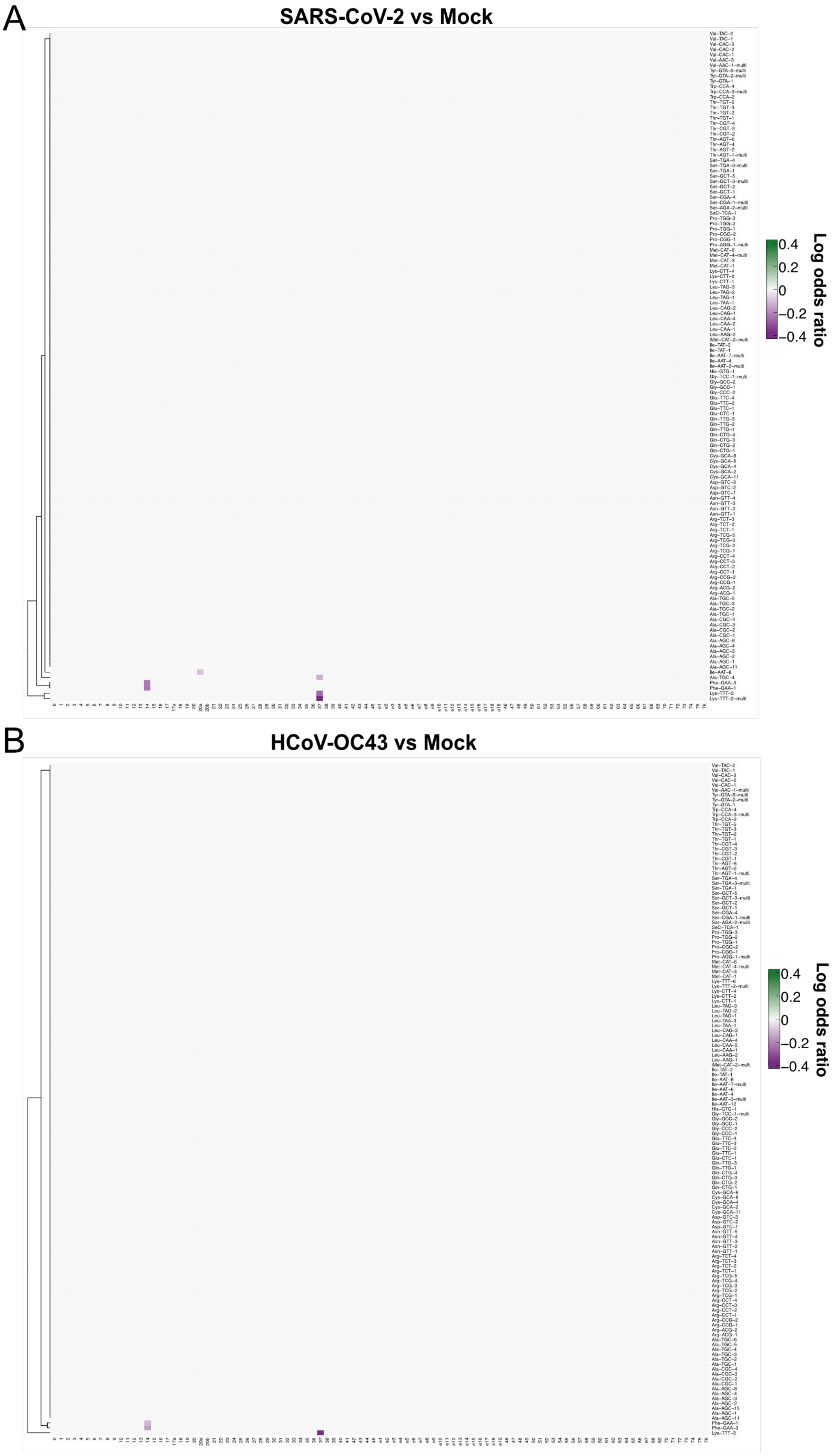

Supplementary Figure 10: tRNA modification analysis in coronavirus infection by mim-tRNA-seq.
Differential modification analysis of SARS-CoV-2 infected Calu3 cells (MOI 3, 32 hours post-infection, n=2) versus Calu3 mock-infected cells (G) and HCoV-OC43 infected MRC5 cells versus MRC5 mock-infected cells (MOI 0.1, 48 hours post-infection, n=3). Heatmaps displays filtered, significant log10 odds ratios (logOR) for each tRNA at each position between mock-infected and infected-cells. Analyses were conducted for all pairwise condition comparisons. Values represent FDR-adjusted chi-squared p-values (≤ 0.01) and are filtered for known and newly detected modified sites in mim-tRNA-Seq.

Supplementary Figure 11

A

	tRNA modifications (N34)		Enzymes	Trigger	Favored Codons
SARS-CoV-2 and HCoV-OC43	mcm^5 and mcm^5s^2	upregulated	KIAA1456 and ALKBH8	DNA damage	Arg-AGA, Gln-CAA, Lys-AAA, Glu-GAA
	I and ^{m}I	downregulated	ADAT2	Infection	Ala-GCU, Arg-CGU, Ile-AUU, Leu-CUU, Pro-CCU, Ser-UCU, Thr-ACU, Val-GUU
	f^5C	downregulated	ALKBH1	oxidative stress	Leu-UUG
HCoV-OC43	Q	upregulated	QTRT1	oxidative stress	Ans-AAU, Asp-GAU, His-CAU, Tyr-UAU

B

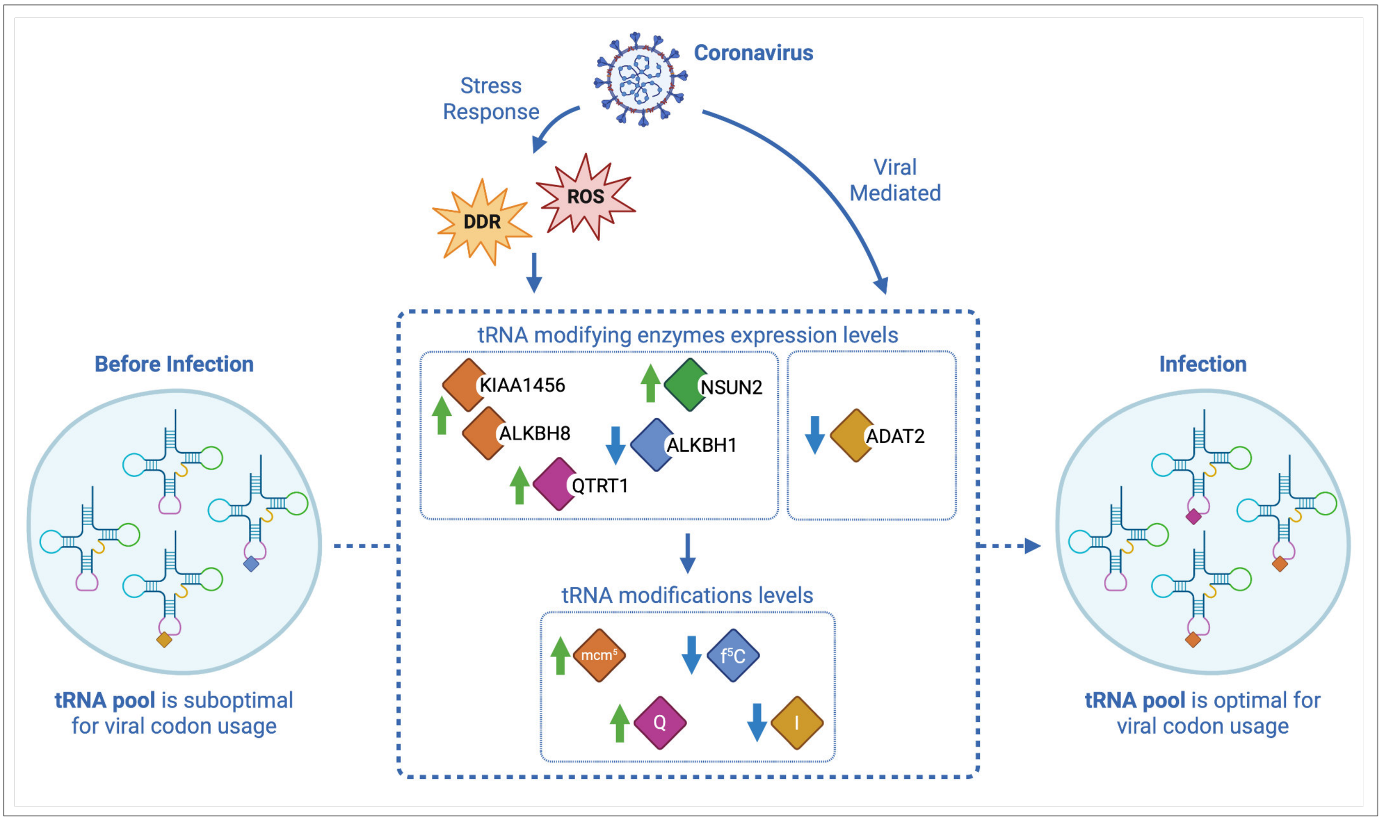

Supplementary Figure 11: Summary Table and Graphical Overview.

(A) Summary table describing the tRNA modifications altered during infection, the enzymes that promote them, the described cellular stress that triggers them, and the codons that are favored. (**B**) Schematic representation of the proposed mechanism by which coronavirus infection reprograms the tRNA modification landscape. Created in BioRender. Diez, J. (2026) https://BioRender.com/llwqhop.

Uncropped Gels Figure 2

2A

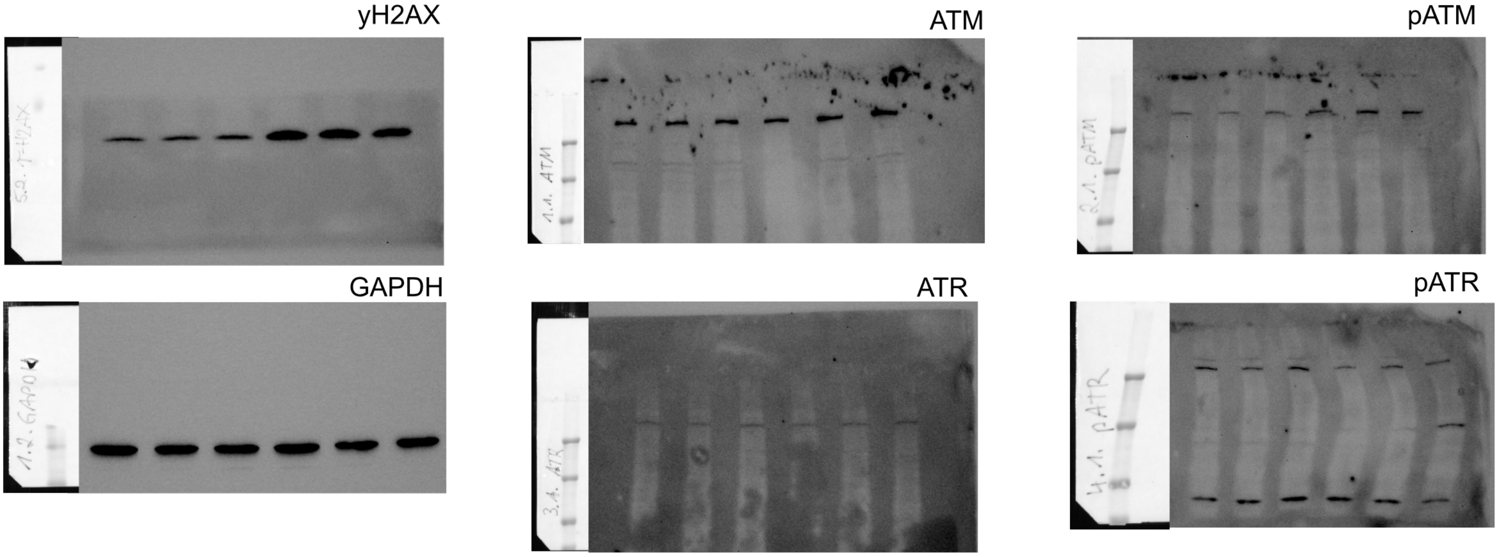

2B

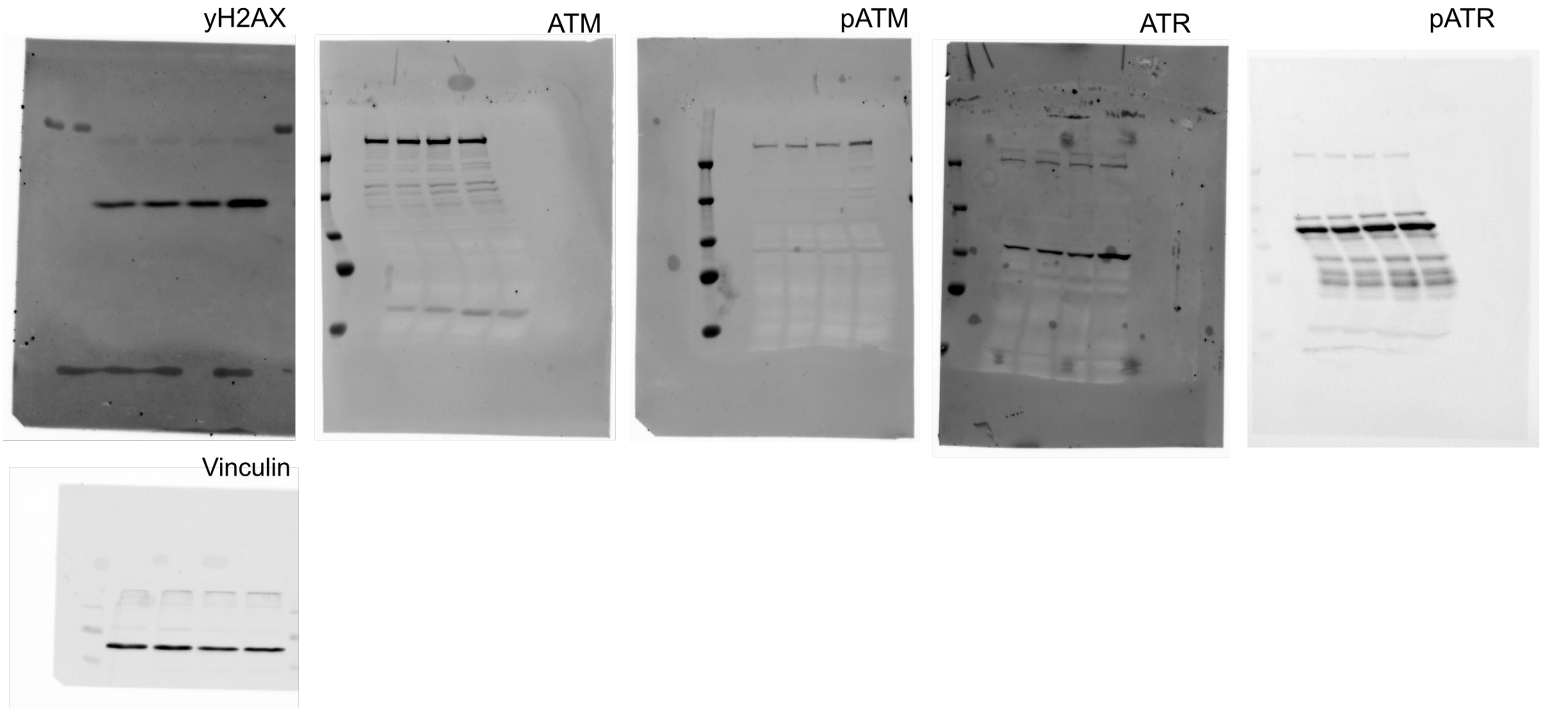

2C

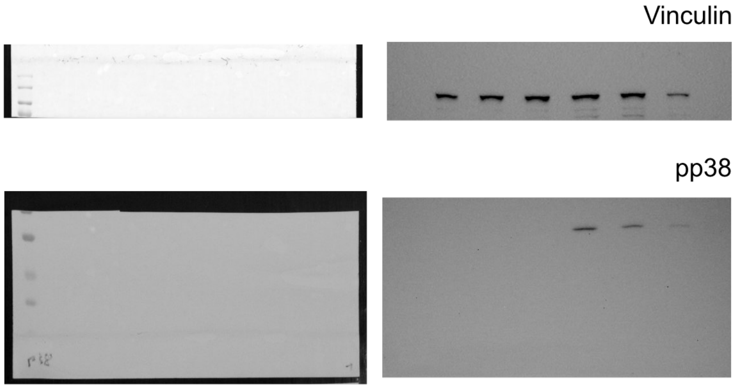

2D

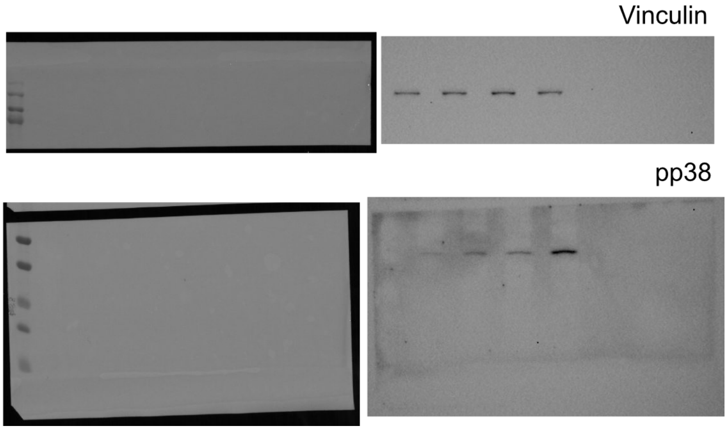

Uncropped Gels Figure 3

3G and 3I - **SARS-CoV-2**

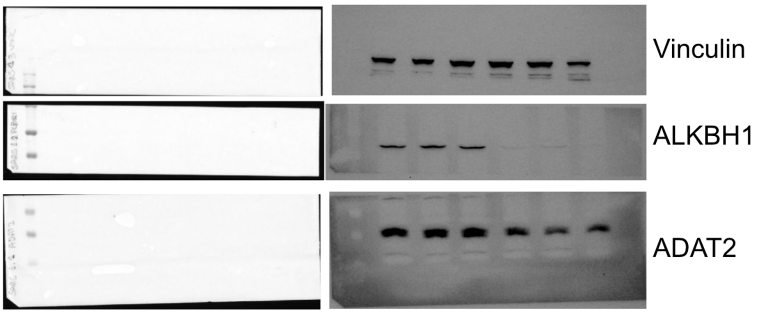

3G and 3I - **HCoV-OC43**

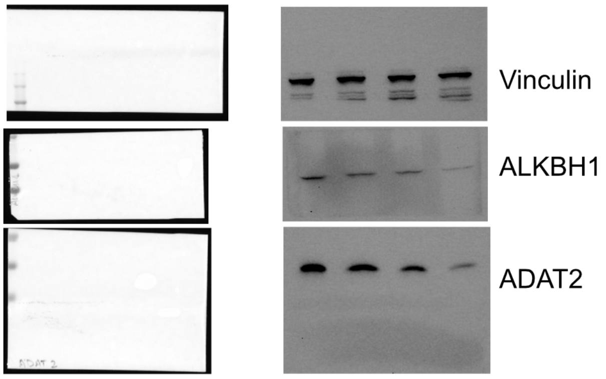

3H - **SARS-CoV-2**

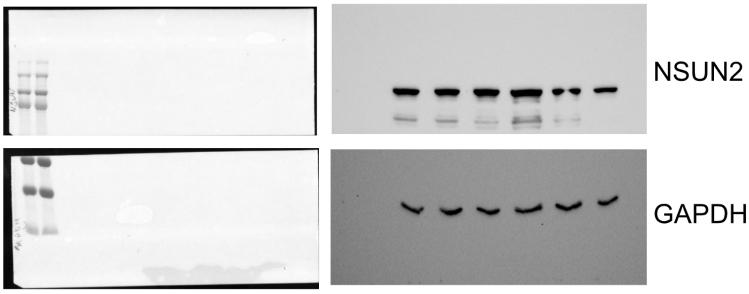

3H - **HCoV-OC43**

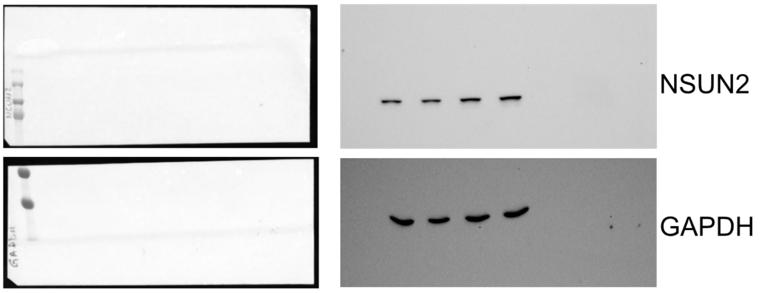

3I- **SARS-CoV-2**

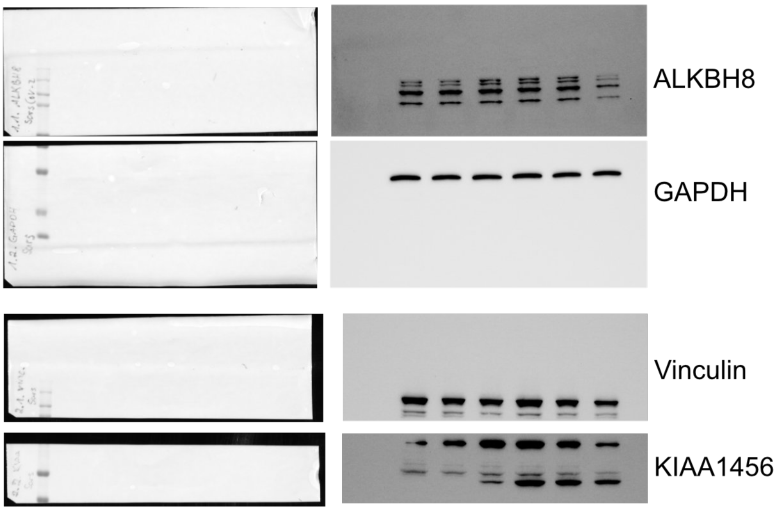

3I- **HCoV-OC43**

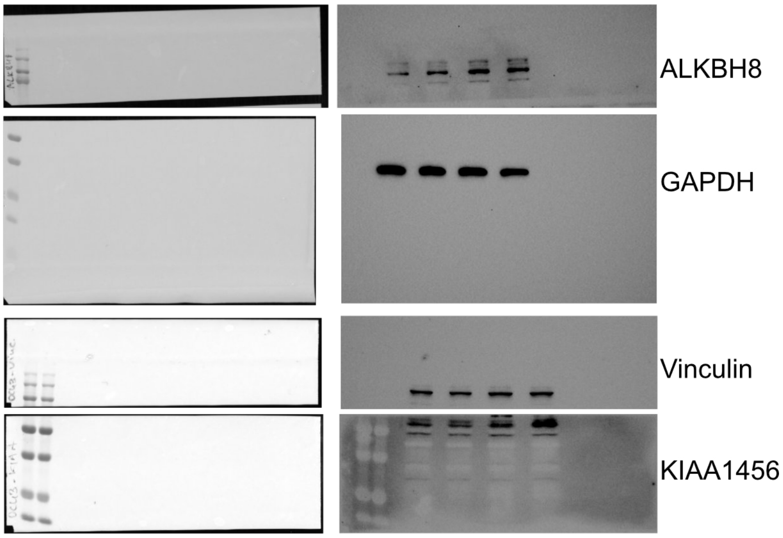

3J - **SARS-CoV-2**

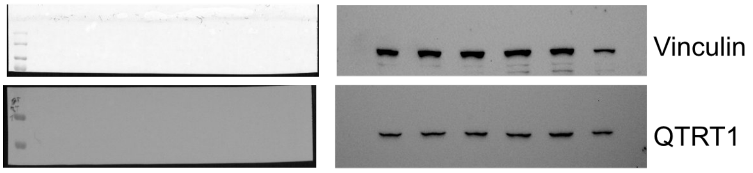

3J - **HCoV-OC43**

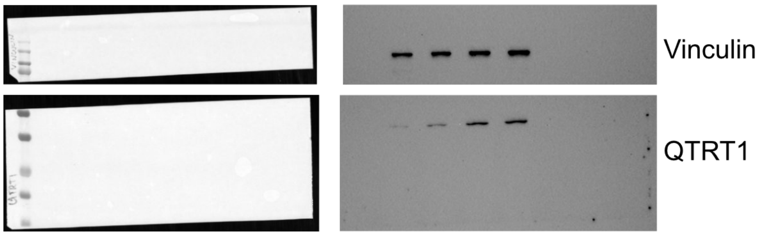

Uncropped Gels Figure 4

4A

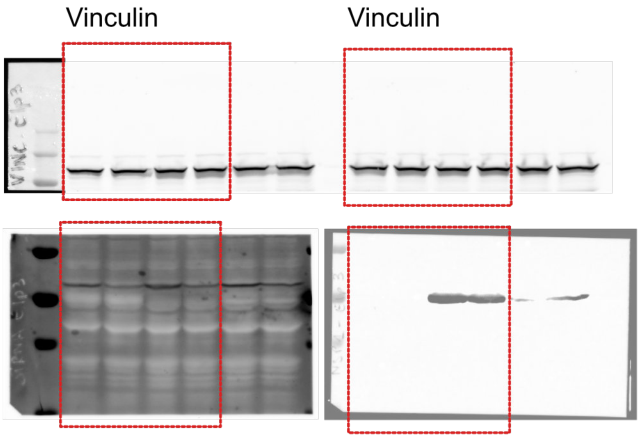

4B

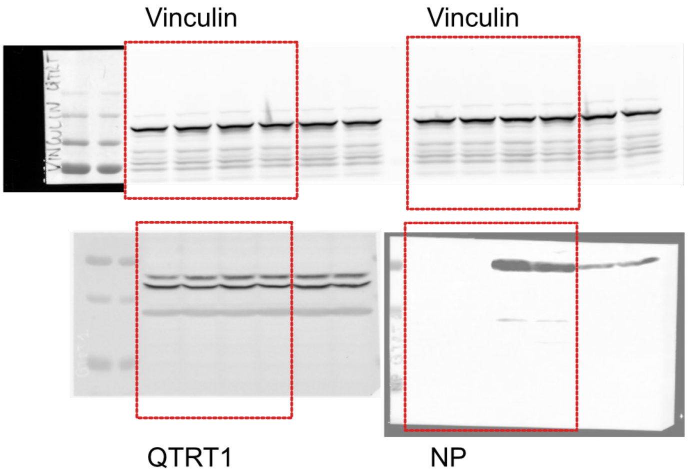

4C

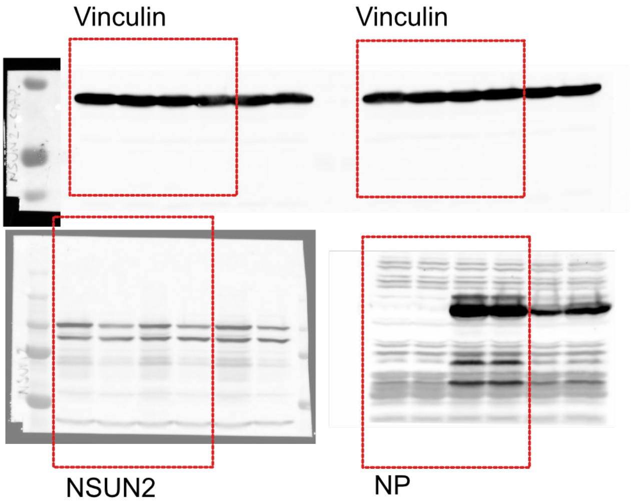

4G

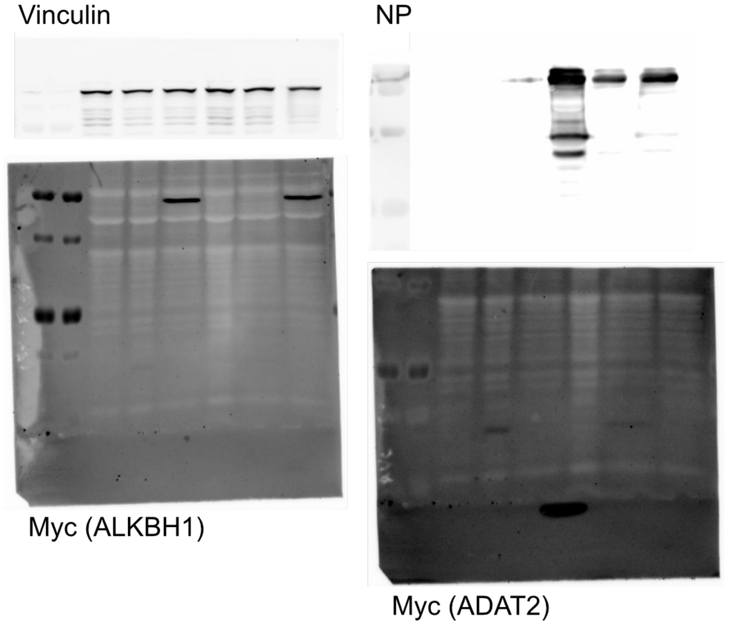

Uncropped Gels Supplementary Figures

Supplementary Figure 2

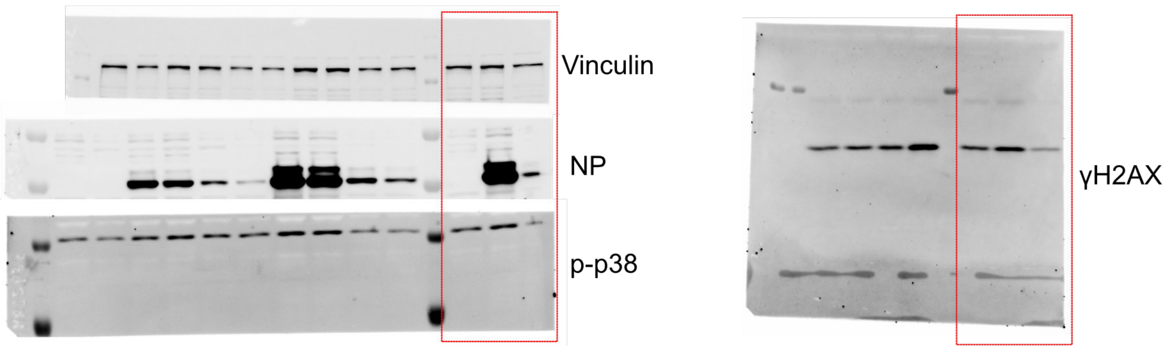

Supplementary Figure 3

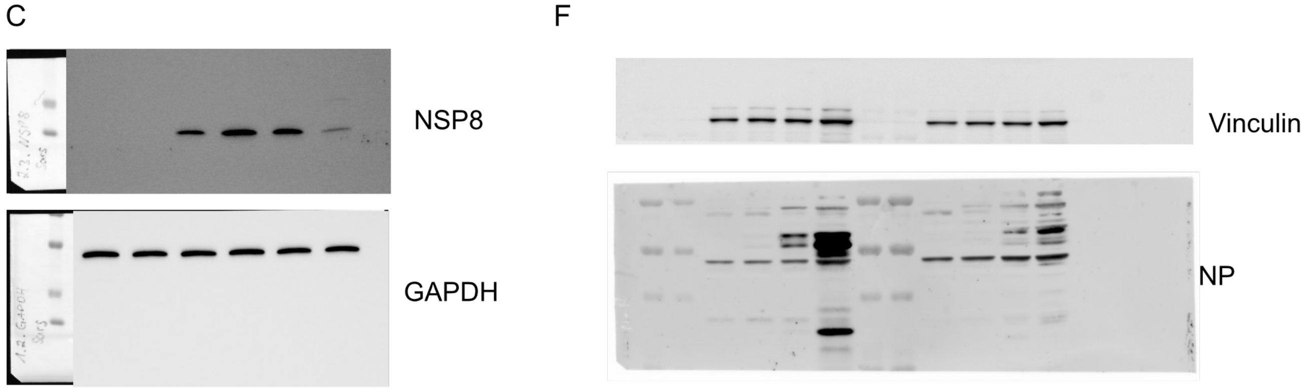

Supplementary Figure 6

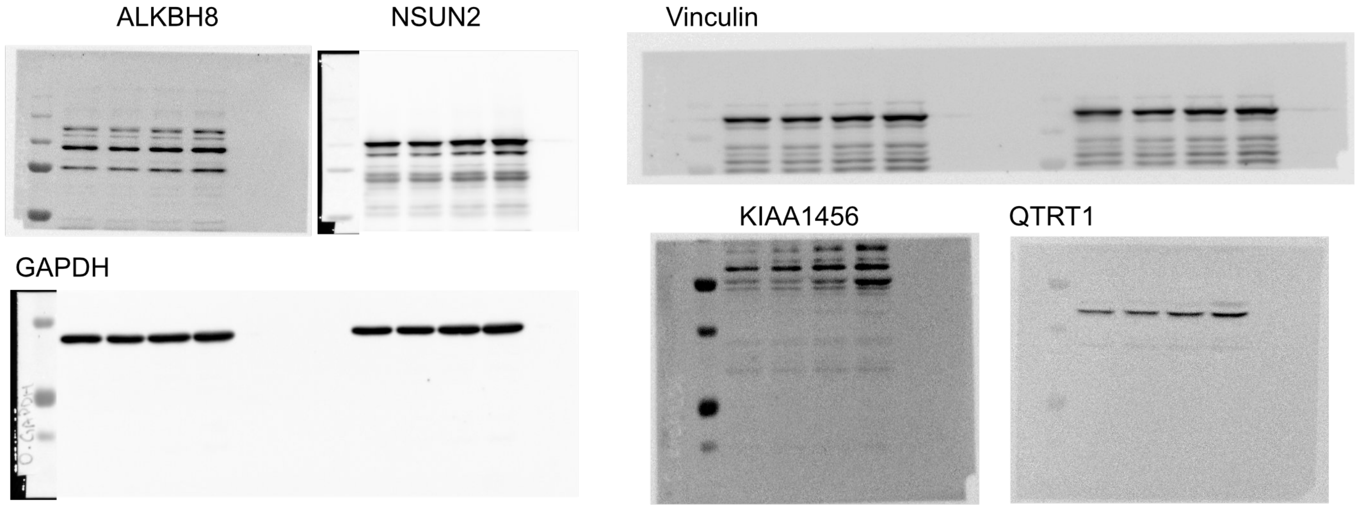

Supplementary Figure 9

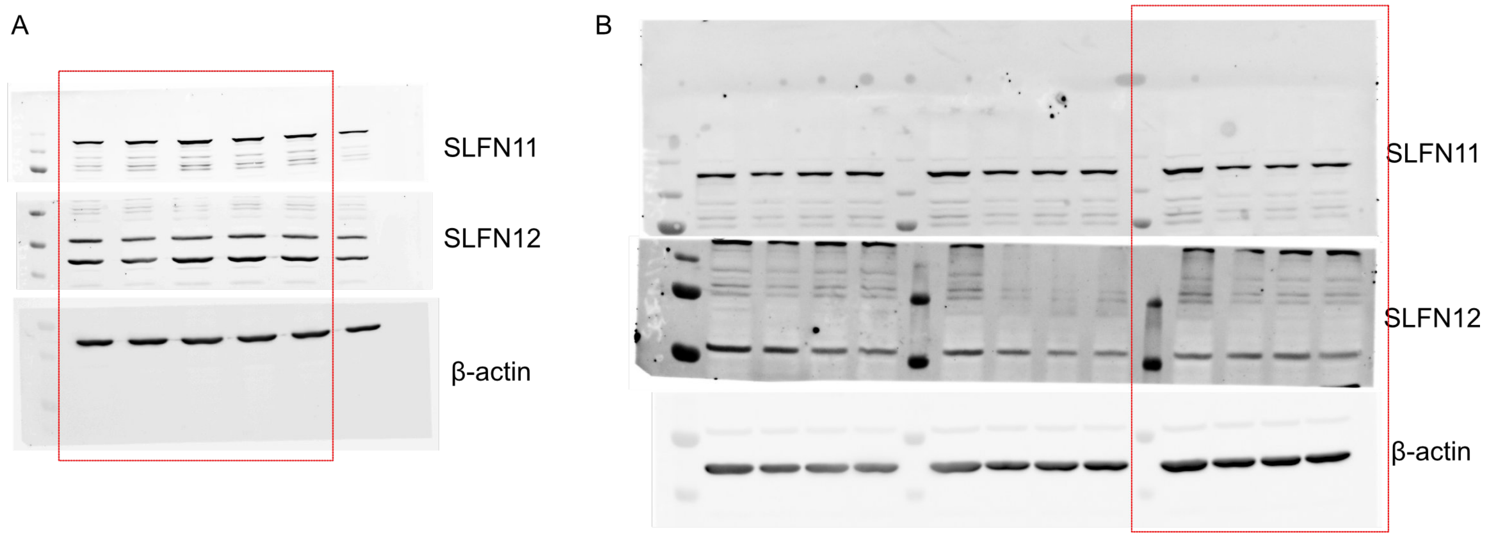

Supplement: Supplementary file 1 — Supplementary Information [file 41467_2026_69700_MOESM1_ESM.pdf]
