# Supplementary material for: Coronaviruses reprogram the tRNA epitranscriptome to favor viral protein expression
Source: Nat Commun. 2026 Feb 19;17:2944. doi: 10.1038/s41467-026-69700-w (PMC13031925; doi:10.1038/s41467-026-69700-w)
Supplement: Supplementary file 2 — Description of Additional Supplementary Files [file 41467_2026_69700_MOESM2_ESM.pdf]

Description of Additional Supplementary Files

Supplementary Data 1

(A) LC-MS/MS tRNA modification list. List of tRNA modifications analyzed by LC–MS/MS. Column 1 reports the abbreviated names of each modification, while Column 2 provides the corresponding full names. **(B) Retention time of the ribonucleoside analyzed.** Retention times and mass spectrometry parameters for the analyzed ribonucleosides. The table includes compound name, m/z, charge state (z), retention time in minutes (start and stop), and HCD collision energy.

Supplementary Data 2

Ribosome profiling and translation efficiency analyses: Results of RNA-seq and Ribo-seq differential expression and translation efficiency analyses. Columns include: gene symbol; RNA-seq base mean expression (baseMean_RNA); RNA-seq log2 fold change (log2FC_RNA); RNA-seq p value (pvalue_RNA); RNA-seq adjusted p value (padj_RNA); mRNA classification (mRNA_class); Ribo-seq base mean expression (baseMean_RPF); Ribo-seq log2 fold change (log2FC_RPF); Ribo-seq p value (pvalue_RPF); Ribo-seq adjusted p value (padj_RPF); translation efficiency in control (TE_control) and SARS conditions (TE_SARS); log2 fold change of translation efficiency (log2FC_TE); adjusted p value for TE (padj_TE); and translation efficiency classification (TE_class).
