# Supplementary material for: Coronaviruses reprogram the tRNA epitranscriptome to favor viral protein expression
Source: Nat Commun. 2026 Feb 19;17:2944. doi: 10.1038/s41467-026-69700-w (PMC13031925; doi:10.1038/s41467-026-69700-w)
Supplement: Supplementary file 3 — Supplementary Data 1 [file 41467_2026_69700_MOESM3_ESM.pdf]

Supplementary Data 1

A) LC-MS/MS tRNA modification list

Modification	Modification full name
C	Cytidine
Y	Pseudouridine
U	Uridine
m3C	3-methylcytidine
m4C	4-methylcytidine
m5C	5-methylcytidine
Cm	2′-O-methylcytidine
A	Adenosine
I	Inosine
m44C	N4,N4-Dimethylcytidine
m4Cm	N4-Methyl-2'-O-methyl-cytidine
5fC	5-Formylcytidine
m5Um	2'-O-Methyl-5-methyluridine
hm5C	5-hydroxymethylcytidine
m1A	1-methyladenosine
m2A	2-methyladenosine
Am	2'O-methyladenosine
m8A	8-methyladenosine
m6A	6-methyladenosine
m1I	1-methylinosine
Im	2′-O-methylinosine
G	Guanosine
ncm5U	5-Carbamoylmethyluridine
cm5U	5-Carboxymethyluridine
m27G	N2,7-Dimethylguanosine
m22G	N2,N2-Dimethylguanosine
mcm5U	5-methoxycarbonylmethyluridine
m227G	N2,N2,7-Trimethylguanosine
mcm5s2U	5-Methoxycarbonylmethyl-2-thiouridine
t6A	N6-threonylcarbamoyladenosine
Q*	Queuosine
manQ/galQ**	Mannosyl-queuosine/Galactosyl-queuosine

Supplementary Data 1

B) Retention time of the ribonucleoside analyzed

			Time (min)		HCD Collision Energy
Compound	m/z	z	start	stop	
C	244.09	1	5.1	15.1	20
U_Y	245.08	1	11.5	32.5	20
Cm_m3C_m4C_m5C_CE20	258.11	1	6	23	20
Cm_m3C_m4C_m5C_CE80	258.11	1	6	23	80
A	268.10	1	23.5	33.5	20
I	269.09	1	24	34	20
5fC	272.09	1	20	35	20
m5Um	273.11	1	33	43	20
hm5C	274.10	1	7	17	20
m1A_m2A_m6A_m8A_Am	282.12	1	5	38	20
Im_m1I	283.10	1	29	39	20
G	284.10	1	24	34	20
ncm5U	302.10	1	18	28	20
cm5U	303.08	1	24	34	20
m27G_m22G	312.13	1	23	42	20
mcm5U	317.10	1	30.5	40.5	20
m227G	326.15	1	23.5	33.5	20
mcm5s2U	333.08	1	36	46	20
Q	410.17	1	15	56	20
t6A	413.14	1	38	48	20
manQ_galQ	572.22	1	15	56	20
