# Supplementary material for: Coronaviruses reprogram the tRNA epitranscriptome to favor viral protein expression
Source: Nat Commun. 2026 Feb 19;17:2944. doi: 10.1038/s41467-026-69700-w (PMC13031925; doi:10.1038/s41467-026-69700-w)
Supplement: Supplementary file 5 — Reporting Summary [file 41467_2026_69700_MOESM5_ESM.pdf]

nature portfolio

Corresponding author(s): Juana Diez

Last updated by author(s): Jan 30, 2026

# Reporting Summary

Nature Portfolio wishes to improve the reproducibility of the work that we publish. This form provides structure for consistency and transparency in reporting. For further information on Nature Portfolio policies, see our Editorial Policies and the Editorial Policy Checklist.

## Statistics

For all statistical analyses, confirm that the following items are present in the figure legend, table legend, main text, or Methods section.

| n/a | Confirmed | |
|---|---|---|
| ☐ | ☒ | The exact sample size (*n*) for each experimental group/condition, given as a discrete number and unit of measurement |
| ☐ | ☒ | A statement on whether measurements were taken from distinct samples or whether the same sample was measured repeatedly |
| ☐ | ☒ | The statistical test(s) used AND whether they are one- or two-sided<br>*Only common tests should be described solely by name; describe more complex techniques in the Methods section.* |
| ☒ | ☐ | A description of all covariates tested |
| ☐ | ☒ | A description of any assumptions or corrections, such as tests of normality and adjustment for multiple comparisons |
| ☐ | ☒ | A full description of the statistical parameters including central tendency (e.g. means) or other basic estimates (e.g. regression coefficient) AND variation (e.g. standard deviation) or associated estimates of uncertainty (e.g. confidence intervals) |
| ☐ | ☒ | For null hypothesis testing, the test statistic (e.g. *F*, *t*, *r*) with confidence intervals, effect sizes, degrees of freedom and *P* value noted<br>*Give P values as exact values whenever suitable.* |
| ☒ | ☐ | For Bayesian analysis, information on the choice of priors and Markov chain Monte Carlo settings |
| ☒ | ☐ | For hierarchical and complex designs, identification of the appropriate level for tests and full reporting of outcomes |
| ☐ | ☒ | Estimates of effect sizes (e.g. Cohen's *d*, Pearson's *r*), indicating how they were calculated |

*Our web collection on statistics for biologists contains articles on many of the points above.*

## Software and code

Policy information about availability of computer code

| | |
|---|---|
| Data collection | No custom software was used. |
| Data analysis | To analyse gel images, these softwares below were used:<br>- Image gauge software FIJI_ImageJ win64 v1.54f<br>- Image Lab (BIO-RAD) version 6.0.1 build 7<br><br>To analyze qPCR, these softwares were used:<br>- qPCR were executed in the QuantStudio™ 3 Real-Time PCR System from ThermoFisher (Cat. #A28567) and data were exported into Microsoft Excel for normalization. A significance test was conducted using a student t-test with GraphPad Prism v10.<br><br>- LC-MS/MS data analysis<br>Aquired data were analyzed with the Skyline-daily software (v24.1.1.284) and extracted precursor areas of the ribonucleosides were used for quantification. Data were exported into Microsoft Excel for normalization. A significance test was conducted using a student t-test with GraphPad Prism v10.<br><br>- Relative synonymous codon usage analysis (RSCU)<br>Sequences were concatenated and RSCU was calculated via the "uco" function from the seqinR package using RStudio (version 2024.09.0 +375).<br><br>- mim-tRNAseq |

Read demultiplexing and adapter trimming were performed with Cutadapt (v3.7). Both read ends were quality-trimmed (-q 30,30), and reads shorter than 10 nt were discarded (-m 10). Indels were not allowed (--no-indels), and only trimmed reads were retained (--trimmed-only). An additional round of trimming was performed to remove two extra 5' nucleotides introduced by cDNA circularization (-u 2). tRNA expression and modification analysis were processed using the mim-tRNAseq computational pipeline (v1.3.8) with the following parameters: --species Hsap --cluster-id 0.97 --threads 5 --min-cov 0.0005 --max-mismatches 0.1 --max-multi 4 --remap --remap-mismatches 0.075.

- Ribosome profiling and RNA-seq analysis
Publicly available ribosome profiling and RNA-seq data from SARS-CoV-2–infected Calu-3 cells (36 hpi; GEO GSE157490) were re-analyzed. Adapters were trimmed with Cutadapt, and reads mapping to non-coding RNAs were removed using Bowtie2. Processed reads were aligned to a combined human (GENCODE v48, MANE Select) and SARS-CoV-2 (NC_045512.2) reference with STAR. Transcript quantification was performed with featureCounts, and ribosome footprint analyses, including A- and P-site assignment, were conducted using riboWaltz. Differential expression and translation efficiency were assessed with DESeq2 and RiboDiff, respectively. Codon usage analysis was performed using seqinR.

For manuscripts utilizing custom algorithms or software that are central to the research but not yet described in published literature, software must be made available to editors and reviewers. We strongly encourage code deposition in a community repository (e.g. GitHub). See the Nature Portfolio guidelines for submitting code & software for further information.

# Data

Policy information about availability of data

All manuscripts must include a data availability statement. This statement should provide the following information, where applicable:

- Accession codes, unique identifiers, or web links for publicly available datasets
- A description of any restrictions on data availability
- For clinical datasets or third party data, please ensure that the statement adheres to our policy

The minimum dataset that supports the findings of this study is provided with this paper. The western blot scans generated in this study are included in the Supplementary Information. The data presented in graphs within the figures are provided in the Source Data File. The ribosome profiling analyses are provided in Supplementary Table 2. The mim-tRNA-seq sequencing data have been deposited in BioProject under the accession number SUB15318744. The mass spectrometry raw data have been deposited in MetaboLights under the accession number MTBLS13738.

The raw mass spectrometry are deposited to the MetaboLights repository with the temporary request number MTBLS13738.

The mim-tRNA-seq datasets from this study have been submitted to the BioProject database with the ID number: PRJNA1262447.

References used for the RSCU analysis are the ones below:
- Human hg38 genome FASTA (https://hgdownload.soe.ucsc.edu/goldenPath/hg38/bigZips/hg38.fa.gz).
- tRNA modifying enzymes sequences were retreived from NCBI GenBank [ ADAT2 (NM_182503.3_cds); ADAT3 (NM_138422.4_cds); ALKBH1 (NM_006020.3_cds); ALKBH8 (NM_001301010.3_cds); KIAA1456 (NM_020844.3_cds); NSUN2 (NM_017755.6_cds); NSUN3 (NM_022072.5_cds); QTRT1 (NM_031209.3_cds); QTRT2 (NM_024638.4_cds).
-Viral coding sequences were retrieved from the NCBI GenBank: SARS-CoV-2 (NC_045512.2_cds); HCoV-OC43 (AY585228.1_cds); Alpha-coronaviruses (NC_023760.1 ; NC_018871.1; NC_028814.1; NC_048216.1; NC_035191.1; NC_030292.1; NC_028833.1; NC_010438.1; NC_048211.1; NC_009657.1; NC_034972.1; NC_076685.1; NC_022103.1; NC_038861.1; NC_046964.1; NC_002645.1; NC_009988.1; NC_054003.1; NC_002306.3; NC_003436.1; NC_054004.1; NC_005831.2; NC_010437.1; NC_028806.1; NC_028752.1; NC_076629.1; NC_055953.1; NC_032107.1; NC_032730.1; NC_028824.1); Beta-coronaviruses (NC_009020.1; NC_004718.3; NC_038294.1; NC_025217.1; NC_019843.3; NC_030886.1; NC_039207.1; NC_006213.1; AC_000192.1; NC_006577.2; NC_009021.1; NC_009019.1; NC_014470.1; NC_045512.2; NC_017083.1; NC_048217.1; NC_003045.1; NC_012936.1; NC_001846.1; NC_026011.1). For alpha and beta coronaviruses, coronavirus genes included ORF1ab, spike, envelope, membrane protein, and nucleocapsid genes, concatenating these sequences in the aforementioned order. Genes such as 3a/b, 4, and 7b were excluded from the study due to annotation inconsistencies.

# Research involving human participants, their data, or biological material

Policy information about studies with human participants or human data. See also policy information about sex, gender (identity/presentation), and sexual orientation and race, ethnicity and racism.

| | |
|---|---|
| Reporting on sex and gender | Our study primarily utilized established cell lines, none of which were derived from primary human or vertebrate tissues. Details regarding the source and sex of these cell lines are provided in the Eukaryotic Cell Lines section.<br><br>For the animal experiments, Golden Syrian hamsters were used, with an equal number of females and males included in the study. |
| Reporting on race, ethnicity, or other socially relevant groupings | NA |
| Population characteristics | NA |
| Recruitment | NA |
| Ethics oversight | Procedures involving animals were performed under UK Home Office License PP0271643 in accordance with the Animals |

(Scientific Procedures) Act 1986 and approved by the University of Glasgow Ethics Committee. All animal research adhered to ARRIVE guidelines.

Note that full information on the approval of the study protocol must also be provided in the manuscript.

# Field-specific reporting

Please select the one below that is the best fit for your research. If you are not sure, read the appropriate sections before making your selection.

- [x] Life sciences
- [ ] Behavioural & social sciences
- [ ] Ecological, evolutionary & environmental sciences

For a reference copy of the document with all sections, see nature.com/documents/nr-reporting-summary-flat.pdf

# Life sciences study design

All studies must disclose on these points even when the disclosure is negative.

| | |
|---|---|
| Sample size | No formal statistical methods were used to predetermine sample size. Sample sizes were selected based on established standards in experimental molecular biology, where a minimum of three independent biological replicates is widely accepted to ensure reproducibility and robustness of the results. In this study, n = 3 biological replicates were used for each condition, and all experiments showed consistent and reproducible trends across replicates. Where fewer replicates were used, this was due to technical limitations, and the observed effects were confirmed by independent experiments or orthogonal methods. The chosen sample size was sufficient to detect biologically meaningful effects and to support the conclusions drawn, in line with common practice in the field. |
| Data exclusions | No data exclusions were performed. |
| Replication | The results were confirmed by performing the experiment multiple times with successful replication (at least in three independent replicates) in conjunction with complementary techniques. |
| Randomization | Samples were allocated randomly for culture, infection, and analysis. |
| Blinding | Blinding was not performed in our study. The experimental parameters determined in this study are considered as objective measures, no subjected to bias and therefore the integrity of the results are not impacted when running the study and analysis unblinded. |

# Reporting for specific materials, systems and methods

We require information from authors about some types of materials, experimental systems and methods used in many studies. Here, indicate whether each material, system or method listed is relevant to your study. If you are not sure if a list item applies to your research, read the appropriate section before selecting a response.

## Materials & experimental systems

| n/a | Involved in the study | |
|---|---|---|
| [ ] | [x] | Antibodies |
| [ ] | [x] | Eukaryotic cell lines |
| [x] | [ ] | Palaeontology and archaeology |
| [ ] | [x] | Animals and other organisms |
| [x] | [ ] | Clinical data |
| [x] | [ ] | Dual use research of concern |
| [x] | [ ] | Plants |

## Methods

| n/a | Involved in the study | |
|---|---|---|
| [x] | [ ] | ChIP-seq |
| [ ] | [x] | Flow cytometry |
| [x] | [ ] | MRI-based neuroimaging |

## Antibodies

Antibodies used

- Anti-SARS-CoV-2 NSP8 [5A10] (GeneTex, GTX632696) mouse monoclonal. Lot number: 42345.
- Anti-KIAA1456 (Thermo Fisher, PA5-70320) rabbit polyclonal. Lot number: ZI4475635.
- Anti-ALKBH8 (Sigma-Aldrich, AV41106) rabbit polyclonal. Lot number: QC21060.
- Anti-ADAT2 (mybiosurce, mbs2521771) rabbit polyclonal. Lot number: AF1823.
- Anti-ALKBH1 [EPR6176] (Abcam, ab126596) rabbit recombinant monoclonal. Lot number: GR3250054-2.
- Anti-NSUN2 (Proteintech, 20854-1-AP) rabbit polyclonal. Lot number: 00122850.
- Anti-QTRT1 (D7) (Santa Cruz Biotechnology, sc398918) mouse monoclonal. Lot number: D1717.
- Anti-Vinculin (Sigma-Aldrich, V9131) mouse monoclonal. Lot number: 0000324945.
- Anti-GAPDH (Proteintech, 60004-1-IG) mouse monoclonal. Lot number: 10017731.
- Anti-Phospho-histone H2AX (©H2AX Ser139) (20E3) (Cell Signaling, 9718S) rabbit monoclonal. Lot number: 21.
- Anti-ATM (D2E2) (Cell Signaling, 2873S) rabbit monoclonal. Lot number: 5.
- Anti-Phospho-ATM (Ser1981) (D25E5) (Cell Signaling, 13050S) rabbit monoclonal. Lot number: 6.
- Anti-ATR (Cell Signaling, 2790S) rabbit polyclonal. Lot number: 10.
- Anti-Phospho-ATR (Ser428) (Cell Signaling, 2853S) rabbit polyclonal. Lot number: 10.

- Anti-Phospho-p38 MAPK (Thr180/Tyr182) (3D7) (Cell Signaling, 9215T) rabbit monoclonal. Lot number: 7.
- Anti-SLFN11 (E-4) (Santa Cruz Biotechnology, sc374339) mouse monoclonal. Lot number: K1623.
- Anti-SLFN12 (Abmart, X-C9J4K7-N) mouse monoclonal combo.
- Anti-HCoV-OC43 Nucleocapsid Antibody (NP) (40643-T62 SinoBiological -abyntech)
- Horseradish peroxidase-conjugated secondary antibodies: anti-mouse (NA931V, lot number: 17212127) and anti-rabbit (NA934V, lot number: 18025286), both from Sigma-Aldrich.
- StarBright Blue 700 Fluorescent Secondary Antibodies: Goat Anti-Mouse IgG (BioRad 12004159) and Goat Anti-Rabbit IgG (BioRad 12004162)

Validation

- Anti-SARS-CoV-2 NSP8 [5A10] (GeneTex, GTX632696) has been validated by the manufacturer in WB, ICC/IF, IHC-Fr, and FCM. This antibody was also validated in previous studies for WB (Sauvat A, Ciccosanti F, Colavita F, et al. On-target versus off-target effects of drugs inhibiting the replication of SARS-CoV-2. Cell Death and Disease. 2020;11(8). doi:10.1038/s41419-020-02842-x). In this study it has been validated for the use in WB.
- Anti-KIAA1456 (Thermo Fisher, PA5-70320) has been validated and published for WB (Jungfleisch J, Böttcher R, Talló-Parra M, et al. CHIKV infection reprograms codon optimality to favour viral RNA translation by altering the tRNA epitranscriptome. Nature Communications. 2022;13(1). doi:10.1038/s41467-022-31835-x). In this study it has been validated for the use in WB.
- Anti-ALKBH8 (Sigma-Aldrich, AV41106) has been validated by the manufacturer in WB. This antibody was also validated in previous studies (Fu D, Brophy J a. N, Chan CTY, et al. Human ALKB homolog ABH8 is a TRNA methyltransferase required for wobble uridine modification and DNA damage survival. Molecular and Cellular Biology. 2010;30(10):2449-2459. doi:10.1128/mcb.01604-09). In this study it has been validated for the use in WB.
- Anti-ADAT2 (mybiosurce, mbs2521771) has been validated by the manufacturer in WB, ELISA, and IHC. In this study it has been validated for the use in WB.
- Anti-ALKBH1 [EPR6176] (Abcam, ab126596) has been validated by the manufacturer in WB, IP, and IHC-P. This antibody was also validated in previous studies (Chen W, Wang H, Mi S, Shao L, Xu Z, Xue M. ALKBH1-mediated m1A demethylation of METTL3mRNA promotes the metastasis of colorectal cancer by downregulating SMAD7 expression. Molecular Oncology. 2022;17(2):344-364. doi:10.1002/1878-0261.13366). In this study it has been validated for the use in WB.
- Anti-NSUN2 (Proteintech, 20854-1-AP) has been validated by the manufacturer in WB, IHC, IF/ICC, FC, IP, CoIP, and ELISA. This antibody was also validated in previous studies (Khoddami V, Cairns BR. Identification of direct targets and modified bases of RNA cytosine methyltransferases. Nature Biotechnology. 2013;31(5):458-464. doi:10.1038/nbt.2566). In this study it has been validated for the use in WB.
- Anti-QTRT1 (D7) (Santa Cruz Biotechnology, sc398918) has been validated by the manufacturer in WB, IP, IF, and ELISA. This antibody was also validated in previous studies (Zhang J, Lu R, Zhang Y, et al. TRNA queuosine modification enzyme modulates the growth and microbiome recruitment to breast tumors. Cancers. 2020;12(3):628. doi:10.3390/cancers12030628). In this study it has been validated for the use in WB.
- Anti-Vinculin (Sigma-Aldrich, V9131) has been validated by the manufacturer in WB, IF, and IHC. This antibody was also validated in previous studies (Gregersen LH, Jacobsen AB, Frankel LB, Wen J, Krogh A, Lund AH. MicroRNA-145 targets YES and STAT1 in colon cancer cells. PLoS ONE. 2010;5(1):e8836. doi:10.1371/journal.pone.0008836). In this study it has been validated for the use in WB.
- Anti-GAPDH (Proteintech, 60004-1-IG) has been validated by the manufacturer in WB, IHC, IF/ICC, FC, IP, CoIP, and ELISA. This antibody was also validated in previous studies (Zhao B, Xu P, Rowlett CM, et al. The molecular basis of tight nuclear tethering and inactivation of cGAS. Nature. 2020;587(7835):673-677. doi:10.1038/s41586-020-2749-z). In this study it has been validated for the use in WB.
- Anti-Phospho-histone H2AX (©H2AX Ser139) (20E3) (Cell Signaling, 9718S) has been validated by the manufacturer in WB, IHC, IF, and FC. This antibody was also validated in previous studies (Pitolli C, Marini A, Guerra M, et al. MYC up-regulation confers vulnerability to dual inhibition of CDK12 and CDK13 in high-risk Group 3 medulloblastoma. Journal of Experimental & Clinical Cancer Research. 2023;42(1). doi:10.1186/s13046-023-02790-2). In this study it has been validated for the use in WB.
- Anti-ATM (D2E2) (Cell Signaling, 2873S) has been validated by the manufacturer in WB. This antibody was also validated in previous studies (Lee JE, Jeon BE, Kwon CS, et al. Norchelerythrine from Corydalis incisa (Thunb.) Pers. promotes differentiation and apoptosis by activating DNA damage response in acute myeloid leukemia. International Journal of Oncology. 2025;66(3). doi:10.3892/ijo.2025.5723). In this study it has been validated for the use in WB.
- Anti-Phospho-ATM (Ser1981) (D25E5) (Cell Signaling, 13050S) has been validated by the manufacturer in WB. This antibody was also validated in previous studies (Mishra S, Krawic C, Luczak MW, Zhitkovich A. Monoubiquitinated H2B, a main chromatin target of formaldehyde, is important for S-Phase checkpoint signaling and genome stability. Molecular Carcinogenesis. 2024;63(12):2414-2424. doi:10.1002/mc.23819). In this study it has been validated for the use in WB.
- Anti-ATR (Cell Signaling, 2790S) has been validated by the manufacturer in WB. This antibody was also validated in previous studies (Nespolo A, Stefenatti L, Pellarin I, et al. USP1 deubiquitinates PARP1 to regulate its trapping and PARylation activity. Science Advances. 2024;10(46). doi:10.1126/sciadv.adp6567). In this study it has been validated for the use in WB.
- Anti-Phospho-ATR (Ser428) (Cell Signaling, 2853S) has been validated by the manufacturer in WB. This antibody was also validated in previous studies (Chen Y, Zhou T, Zhou R, et al. TRAF7 knockdown induces cellular senescence and synergizes with lomustine to inhibit glioma progression and recurrence. Journal of Experimental & Clinical Cancer Research. 2025;44(1). doi:10.1186/s13046-025-03363-1). In this study it has been validated for the use in WB.
- Anti-Phospho-p38 MAPK (Thr180/Tyr182) (3D7) (Cell Signaling, 9215T) has been validated by the manufacturer in WB, IF, and FC. This antibody was also validated in previous studies (Lee D, Jeon J, Baek S, et al. CycloZ suppresses TLR4-Driven inflammation to reduce asthma-like responses in HDM-Exposed mouse models. Cells. 2024;13(23):2034. doi:10.3390/cells13232034). In this study it has been validated for the use in WB.
- Anti-SLFN11 (E-4) (Santa Cruz Biotechnology, sc374339) has been validated by the manufacturer in WB, IP, IF, IHC(P), and ELISA. This antibody was also validated in previous studies (Waters T, Goss KL, Koppenhafer SL, Terry WW, Gordon DJ. Eltrombopag inhibits the proliferation of Ewing sarcoma cells via iron chelation and impaired DNA replication. BMC Cancer. 2020;20(1). doi:10.1186/s12885-020-07668-6). In this study it has been validated for the use in WB.
- Anti-SLFN12 (Abmart, X-C9J4K7-N) has been validated by the manufacturer in ELISA. In this study it has been validated for the use in WB.
- Horseradish peroxidase-conjugated secondary antibodies (NA931V, lot number: 17212127) and anti-rabbit (NA934V, lot number: 18025286) were validated by the manufacturer.

- StarBright Blue 700 secondary antibodies were validated by the manufacturer.
- HCoV-OC43 Nucleocapsid Antibody (NP) (40643-T62 SinoBiological -abyntech) has been validated by the manufacturer for Wester Blot and ELISA.

# Eukaryotic cell lines

Policy information about cell lines and Sex and Gender in Research

| | |
|---|---|
| Cell line source(s) | - MRC5 (kindly provided by Wolfram Brune), male<br>- Calu3 (kindly provided by Alfredo Castello), male.<br>- A549 (kindly provided by Ana Janic), male |
| Authentication | Cell lines were not authenticated for this study. |
| Mycoplasma contamination | All cell lines were tested negative for mycoplasma contamination. |
| Commonly misidentified lines (See ICLAC register) | No commonly misindentified cell lines were used. |

# Animals and other research organisms

Policy information about studies involving animals; ARRIVE guidelines recommended for reporting animal research, and Sex and Gender in Research

| | |
|---|---|
| Laboratory animals | Golden Syrian Hamsters |
| Wild animals | NA |
| Reporting on sex | Golden Syrian hamsters were used in this study, with an equal number of male (n=3, for condition) and female (n=3, for condition) animals included in the experimental design. However, sex was not considered a relevant biological variable for the objectives of this study, as no sex-specific effects were anticipated based on the nature of the experimental intervention and the endpoints measured. |
| Field-collected samples | NA |
| Ethics oversight | Procedures involving animals were performed under UK Home Office License PP0271643 in accordance with the Animals (Scientific Procedures) Act 1986 and approved by the University of Glasgow Ethics Committee. All animal research adhered to ARRIVE guidelines. |

Note that full information on the approval of the study protocol must also be provided in the manuscript.

# Plants

| | |
|---|---|
| Seed stocks | NA |
| Novel plant genotypes | NA |
| Authentication | NA |

# Flow Cytometry

## Plots

Confirm that:

- [x] The axis labels state the marker and fluorochrome used (e.g. CD4-FITC).
- [x] The axis scales are clearly visible. Include numbers along axes only for bottom left plot of group (a 'group' is an analysis of identical markers).
- [x] All plots are contour plots with outliers or pseudocolor plots.
- [x] A numerical value for number of cells or percentage (with statistics) is provided.

## Methodology

| | |
|---|---|
| Sample preparation | Intracellular reactive oxygen species (ROS) were quantified in HCoV-OC43–infected A549 cells using the fluorescent probe DCFH-DA (Sigma-Aldrich, D6883). Cells were infected at an MOI of 0.1 and incubated for 48 h at 33 °C, then stained with 10 µM DCFH-DA for 30 min at 37 °C in the dark. Cells were then trypsinized and resuspended in phenol red–free medium.. |
| Instrument | Fluorescence was measured in the FITC channel (488 nm) using a BigFoot Spectral Cell Sorter (ThermoFisher Scientific). |
| Software | Mean fluorescence intensity (MFI) was calculated with FlowJo (BD Biosciences) to determine relative ROS levels |
| Cell population abundance | A total of 20,000 events per sample were acquired and analyzed by flow cytometry; no cell sorting was performed. |
| Gating strategy | The gating strategy included initial selection of cells based on forward scatter (FSC-A) and side scatter (SSC-A) to exclude debris, followed by gating on FSC-H vs FSC-A to remove doublets. ROS levels were quantified within the singlet live-cell population by measuring FITC fluorescence (488 nm) corresponding to DCFH-DA signal intensity. Mean fluorescence intensity (MFI) was calculated for each condition, with unstained samples used to define background fluorescence. |

☒ Tick this box to confirm that a figure exemplifying the gating strategy is provided in the Supplementary Information.
